# Endothelial cell, but not neutrophil, programmed cell death receptor-ligand 1 loss has a morbid impact on experimental murine shock/sepsis-induced lung injury

**DOI:** 10.3389/fimmu.2026.1816915

**Published:** 2026-06-02

**Authors:** Elizabeth W. Tindal, Chun-Shiang Chung, Yaping Chen, Runping Zhao, Fernando Gutierrez Garcia, Theopi Rados, Sean F. Monaghan, Alfred Ayala

**Affiliations:** Division of Surgical Research, Department of Surgery, Brown University Health-Rhode Island Hospital/Brown University, Providence, RI, United States

**Keywords:** endothelial cells, indirect-acute lung injury, mice, neutrophils, PD-L1

## Abstract

**Introduction:**

Our laboratory and others have shown that programmed cell death receptor-ligand 1 (PD-L1) contributes to the development of shock/sepsis-induced morbidity and mortality, but its role appears to vary across organ/cell type.

**Objective:**

Here, we leverage the construction of Cre-lox mouse models to produce mice constitutively lacking either PD-L1 gene expression on endothelial cells (*ec*PD-L1^−/−^) or neutrophils (*pmn*PD-L1^−/−^) to test the hypothesis that endothelial cell as opposed to neutrophil deficiency of PD-L1 differentially contributes to shock/sepsis-induced lung injury/death. Methods: Adult male C57BL/6 (breeder background strain), *ec*PD-L1^−/−^, *pmn*PD-L1^−/−^, and/or mixed ^flox^ (*ec* and *pmn* PD-L1^−/+^ or PD-L1^+/+^, respectively)-no Cre (Control) mice were subjected to either hemorrhagic (Hem) shock followed 24 h by cecal ligation and puncture (CLP) (Hem/CLP) or sham Hem and sham CLP (Sham). Survival studies were performed, and a separate set of animals were taken at 24 h post-procedure for peripheral blood, bronchoalveolar lavage fluid (BALF), and lung tissue collection. Samples were processed and stained for analysis by flow cytometry, cytokine, chemokine, and angiopoietin ELISAs and indices of organ injury assays. A subset of animals was also examined for changes in lung permeability using Evan’s Blue dye exclusion.

**Results:**

Fourteen-day mortality in the *ec*PD-L1^−/−^ mice was lower than in the Hem/CLP Control group, while the mortality rate was increased in the *pmn*PD-L1^−/−^ vs. Controls. Lung vascular permeability was also markedly decreased in the *ec*PD-L1^−/−^ Hem/CLP mice, but no such decline was seen in the lungs of *pmn*PD-L1^−/−^ mice. While Hem/CLP increased the lung tissue, BALF, and blood levels of several cytokine, chemokine, and angiopoietin levels, the concentrations of lung tissue and BALF MCP-1 and blood urea nitrogen markedly declined in the *ec*PD-L1^−/−^ vs. Control mice. Alternatively, the lung levels of Angiopoietin 2 and BALF MIP-2 and IL-6 concentrations significantly increased in Hem/CLP *pmn*PD-L1^−/−^ animals.

**Conclusions:**

Taken together, these results support the hypothesis we have previously proffered, that expression of PD-L1 on endothelial cells has a morbid impact. However, surprisingly, we have also uncovered a potential immune protective role of PD-L1 expression on neutrophils.

## 1 Introduction

Traumatic injury, of which rapid and excessive blood loss is often a significant component, remains a major concern for healthcare providers worldwide as it is highly associated with clinical morbidities (1–4). Following traumatic injury, the timing available to clinicians for rapid treatment and resuscitation is tightly linked to patient survival, and while trauma care directed toward rapid patient stabilization and support continues to advance, there remain a variety of comorbidities concurrent with hypovolemic shock that still present significant challenges to successful treatment (5, 6). Of these morbidities, the development of sepsis is particularly detrimental to survival. “Sepsis” is now defined as a life-threatening organ dysfunction caused by a dysregulated host response to infection (Sepsis 3) (7). Relative to the host response to injury/infection, sepsis is proposed to result from a multi-factorial disharmony of immune cell function, in which circulating circulating leukocytes influx into distal organs, contributing directly or indirectly to tissue and organ injury (8, 9). Most frustrating is that while we continue to optimize the supportive care for these critically ill patients, we have yet to see a novel molecular etiology-based therapy make a sustained impact on overall septic morbidity and mortality (10, 11).

Lung injury can be the result of a “primary/direct” pulmonary process that has its derivation from the lung such as pneumonia, to aspiration or some other direct injury issue to the lung itself, or it can be an “indirect” result from a distal inflammatory and/or infectious process located elsewhere in the body as is the case in traumatic shock/injury, non-pulmonary sepsis, pancreatitis, etc. The most severe form of either is defined as acute respiratory distress syndrome (ARDS) and presents with hypoxemia and radiological infiltrates, fluid buildup, and edema on chest x-ray (12). Unfortunately, the pathomechanisms leading to the development of “indirect” acute lung injury (ALI)/ARDS (13) remain elusive. However, two theories have been proposed based on our current understanding: first, that “primed” neutrophils migrate to the lung, where they reside until subsequently triggered to release their bactericidal and inflammatory cargos, when triggered by a secondary stimulus, culminating in “by-stander” lung tissue damage (14–17). The second proposes that local pulmonary immune cell activation predisposes epithelial and endothelial cell dysfunction that drives iARDS (17–20).

That said, while we have defined numerous defects of components of both adaptive and innate immune responsiveness induced by experimental shock and/or sepsis, our laboratory has uncovered novel role(s) for a number of the members of the B7-family of cell-associated co-inhibitory receptors, i.e., programmed cell death receptor-1 (PD-1), B-/T-lymphocyte attenuator (BTLA), and, recently, V-domain immunoglobulin suppressor of T-cell activation (VISTA, a.k.a., B7-H5 and PD-1H) and their respective cell surface ligands, popularly referred to as checkpoint proteins, which appear to contribute to aspects of organ and/or lung injury (21). Classically, the function of PD-1 and PD-L1 has been defined based on their function or interactions with T cells during the process of antigen presentation and subsequent T-cell activation. After the first signal through the major histocompatibility complex, the T cell requires a second signal, a sort of stop or go signal in the form of these immune checkpoint proteins that serve to co-stimulate or co-inhibit its activation. Previously, we have reported that in a model of traumatic shock and subsequent septic challenge, as produced using experimental hemorrhage and cecal ligation and puncture (CLP), global PD-L1 knockout mice had significantly improved survival when compared to their wild-type counterparts (22). Furthermore, these mice did not develop acute lung injury as evidenced by their protein levels in bronchoalveolar lavage fluids (BALFs) and maintained endothelial integrity in the lung tissue through stable expression of VE-cadherin. Lastly, we saw a reduction in lung tissue cytokine levels including TNF-α, IL-6, and MCP-1. Considering these findings altogether, we speculated that PD-L1 expression on endothelial cells might have a negative impact (an increase) on lung permeability, related to a reduction in supportive endothelial growth factors and loss of junctional protein integrity. Systemically, increased PD-L1 expression appears to be associated with increased organ damage, a rise in cytokine release, and death in response to the combined insults of shock and sepsis. However, while leaning toward a pathological role for PD-L1-induced expression by endothelial cells in the morbidity/mortality that has been previously reported (23), the approaches in these studies cannot rule out a role for PD-L1 expression on leukocytes like neutrophils.

To arrive at the question of how does expression of PD-L1 on endothelial cells as opposed to leukocytes, specifically neutrophils, affect the onset of shock/septic lung iALI/iARDS and/or overall morbidity/mortality, we chose to construct and compare an endothelial cell lineage as opposed to neutrophil cell lineage-specific mice that are constitutively deficient for the PD-L1 gene. In other words, we wanted to test the hypothesis that endothelial cell, but not neutrophil, restricted loss of PD-L1 gene expression provides protection from shock/sepsis-induced iARDS and/or overall morbidity/mortality.

## 2 Materials and methods

### 2.1 Mice

**Figure 1:**
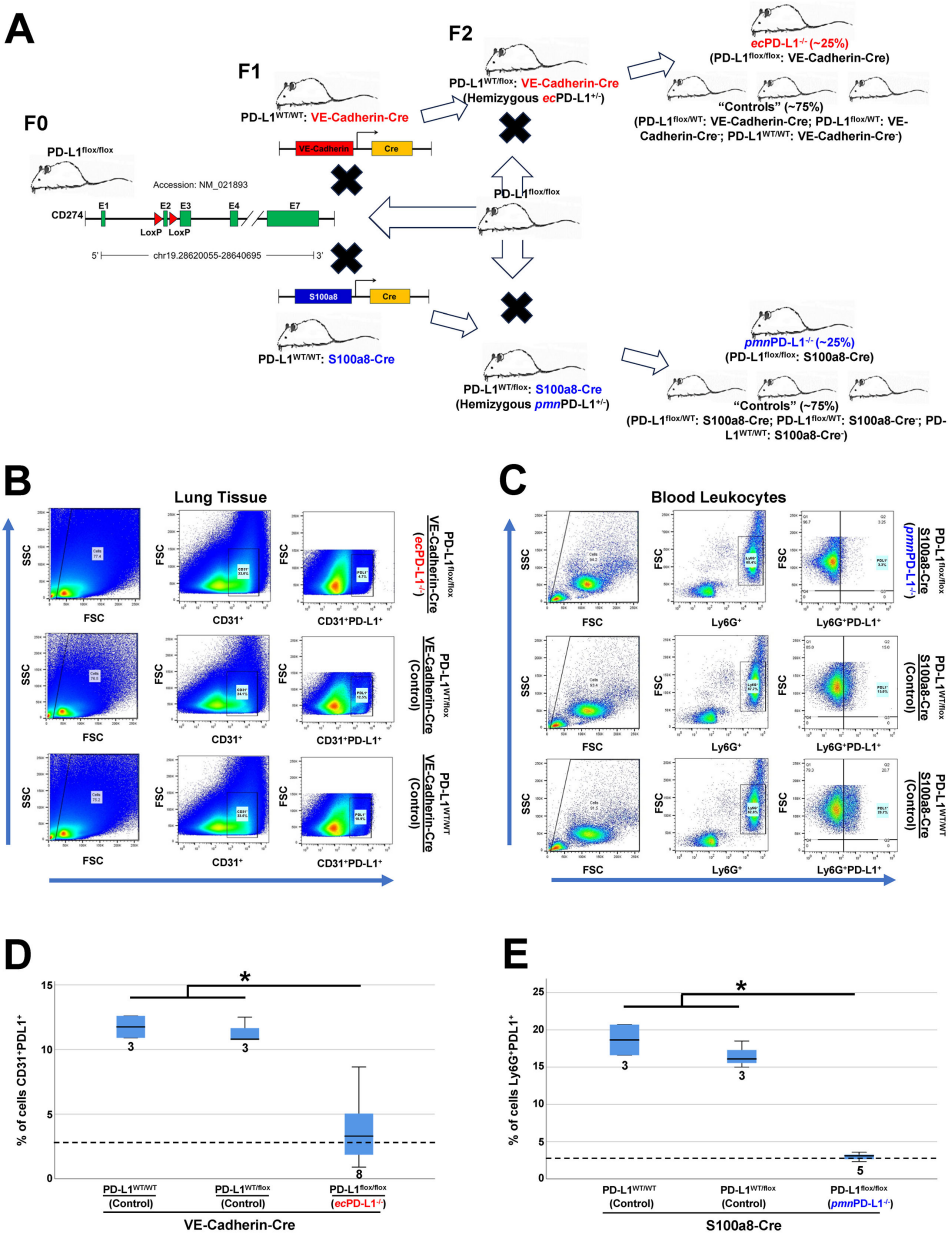
Description **(A)** and characterization of endothelial cell (*ec*PD-L1^−/−^) **(B, D)** or neutrophil (*pmn*PD-L1^−/−^) **(C, E)** restricted constitutive PD-L1 gene-deficient mice. Taconic Knockout Repository [mouse TF0103 (MGI:1926446); Taconic Biosciences Inc., Germantown, NY], CD274/PD-L1 conditional knockout mouse. PD-L1 gene exons 1 to 4 are shown as green boxes and loxP sites flanking exon 2 are depicted as red triangles **(A)**. Homozygous PD-L1^flox/flox^ were subsequently crossed to either VE-Cadherin‐Cre (B6.FVB-Tg[Cdh5-cre]7Mlia/J; Strain #:006137; RRID: IMSR_JAX:006137) or the S100a8-Cre (B6.Cg-Tg[S100a8-cre,-EGFP]1IIw/J; Strain #:021614; RRID: IMSR_JAX:021614) mice, obtained from Jackson Labs, to produce either mice homozygous for both the ^flox^ PD-L1 and specific cell-lineage Cre promoter genes or mice that were heterozygous for one or both genes that served as “Controls”. Gating strategy and typical flow cytogram/dot plot of select cell-lineage restricted PD-L1 expression [either CD31 and PD-L1 **(B)** or Ly6G and PD-L1 **(C)**] for various breeding outcomes from respective Cre-Lox matings for PD-L1^flox/flox^ animals with VE-Cadherin‐Cre **(B)** or PD-L1^flox/flox^ animals with S100a8-Cre **(C)**. Summary data for the change in percentage PD-L1^+^ endothelial cells (CD31^+^) **(D)** and percentage PD-L1^+^ neutrophils (Ly6G^+^) **(E)**. A dotted line is provided to indicate median frequency of non-specific antibody binding typically detected using gating strategy during analysis, which was 2.75% ± 1.77% CD31^+^PD-L1^+^ **(D)** and 2.29 ± 0.50% Ly6G^+^PD-L1^+^ **(E)**, respectively. The presence of a significant difference between groups was established at *p < 0.05 with a Mann–Whittney *U* test.

Male C57BL/6 mice were purchased from The Jackson Laboratory (Bar Harbor, ME). Animals obtained from our outside vendor were acclimated no less than 7 days, and often longer (maximum ~5 weeks), prior to utilizing these animals in the studies described here. During this period, they were housed in the Rhode Island Hospital (RIH) rodent facility (12-h:12-h light/dark cycle, 23–25 °C, 30%–70% humidity) where they received standard care and diet (standard rodent chow)/water *ad libitum*. All protocols were carried out in the morning (8–11 a.m.) and were performed in accordance with the National Institutes of Health guidelines and as approved by the Animal Use Committee of Rhode Island Hospital (AWC# 5054–21 and 5028-24). PD-L1[CD274/B7-H1]^loxP^ mouse (PD-L1^flox/flox^) was derived from cryopreserved embryo at the Taconic Knockout Repository [mouse TF0103 (MGI:1926446)] at the Taconic Biosciences Inc. (Germantown, NY), which had LoxP sites inserted by targeted mutation at chromosome 19.28620055–28640695 gene NM_021893 (**Figure 1A**). The VE-Cadherin‐Cre (Strain #:006137; RRID: IMSR_JAX:006137) and the S100a8-Cre (Strain #:021614; RRID: IMSR_JAX:021614) breeder mouse strains were also obtained from The Jackson Laboratory (see **Figure 1A**).

### 2.2 Endothelial cell or neutrophil restricted constitutive PD-L1 gene-deficient mice

Initial breeding strategy for the construction of Cre-lox mouse used in these studies is laid out in **Figure 1A**. Initially, we established an F0 colony of homozygous PD-L1^flox/flox^ [no Cre transgene] mice. Subsequently (F1), “Hemizygous” mice were derived from a cross of PD-L1^flox/flox^ [no Cre transgene] mice with select Cre transgene containing mice that are PD-L1^WT/WT^. Then, finally (F2), the PD-L1^flox/flox^:VE-Cadherin‐Cre mice (*ec*PD-L1**^−^**^/^**^−^**) and the PD-L1^flox/flox^:S100a8-Cre mice (*pmn*PD-L1**^−^**^/^**^−^**) (full Cre-Lox constructs; typically ~25% of progeny), respectively, as well as the resultant (~75% of the progeny) PD-L1^WT/flox (+/−)^ or PD-L1^WT/WT (+/+)^ animals with/without a Cre transgene (which served as Cre-Lox mouse “Controls”) used in the experimental protocol were products of back-cross of the F0 PD-L1^flox/flox^ homozygous breeder mice with F1 “Hemizygous” mice. Routine genotyping of the PD-L1 gene expression, with [^WT^ or ^WT/flox^]; without the LoxP insertion [^flox/flox^]; the VE-Cadherin‐Cre transgene; and S100a8-Cre transgene mice was performed on tail biopsy samples collected after weaning (**Supplementary Figures 1**, **2**) (24). In brief, tail samples (0.3–0.5 cm) were processed for PCR (primers described in **Supplementary Table 1**) in 100 µL of Direct PCR Lysis Reagent (Viagen Biotech, Inc. Cat # 101-T) and incubated at 85°C for 45 min, and the DNA was spun down before combining in 2× PCR Master Mix (Promega: #M7502) containing 100 µM of DNA oligos of interest from Integrated DNA Technologies (Coralville, IA). Following PCR amplification (amplification cycles: initial step, 5 min at 95°C; 30 cycles of 1 min at 95°C/1 min at 60°C/1 min at 72°C; 5 min at 72°C) with SimpliAmp Thermal Cycler (Thermo Fisher Scientific, Waltham, MA), samples were run on a 1% agarose gel containing ethidium bromide and imaged for gene deletion analysis and validation using a ChemiDoc Imaging System (Bio-Rad Laboratories, Inc., Hercules, CA). Male mice with appropriate base pair deletion were used for downstream studies (25). PCR products were detected using primer pairs synthesized by Integrated DNA Technologies for the PD-L1 gene as described/designed by Taconic Biosciences Inc. and for both VE-Cadherin‐Cre transgene and S100a8‐Cre transgene as described/designed by The Jackson Laboratory (see **Supplementary Table 1**).

Flow cytometric analysis of single-cell lung tissue specimens or the analysis of peripheral blood leukocytes isolated from either “genotyped” *ec*PD-L1**^−^**^/^**^−^**, *pmn*PD-L1**^−^**^/^**^−^**, or their respective “Control” animals was also performed to document the PD-L1 endothelial cell restricted phenotypic deficiency or the PD-L1 neutrophil restricted phenotypic deficiency of either the *ec*PD-L1**^−^**^/^**^−^** or *pmn*PD-L1**^−^**^/^**^−^** animals, respectively, as compared to “Control” mice (**Figures 1B–E**) (22, 25). We also observed no evidence of gene dosage/partial penetrance effect within the “Control” mouse specimens, which is in keeping with what is reported for the PD-L1 (**Figures 1D, E**) (26). Details of the actual staining protocol for flow cytometric analysis are described below.

### 2.3 Hemorrhage/CLP model

A model of hypovolemic shock (Hem) was implemented, whereby a fixed-pressure hemorrhage protocol was utilized to achieve a sustained reduced mean arterial blood pressure in C57BL/6 (8–12 weeks old), various in-house bred incomplete floxed-Cre background control (Control), or *ec*PD-L1**^−^**^/^**^−^** or *pmn*PD-L1**^−^**^/^**^−^** 8- to 12-week-old male mouse hosts (27). This choice was made so as to maximize our ability to initially see an experimental difference in the ALI/ARDS response based on previous reports that male mice did poorer in response to these experimental stressors of shock (hemorrhage) and/or septic challenge than pro-estrus stratified females (28–31). In brief, an isoflurane/oxygen gas mixture was used as anesthesia under the RIH IACUC-approved protocol for animal safety (AWC# 5054–21 and 5028-24). Bilateral arteriotomies were catheterized and used to monitor blood pressure and draw blood throughout the procedure. Mice were kept in this state for a 90-min duration, during which additional blood was drawn to maintain reduced blood pressure ~40 mmHg (±5 mmHg). This provides a standardized and effective mimic of severely injured hypovolemic patients. Immediately following this experimental insult, mice were administered a crystalloid solution of lactated ringer’s equivalent to 4× the volume of blood hemorrhaged. Sham surgeries were performed under anesthesia by which both femoral arteries were ligated. However, blood was not drawn, and these serve as negative controls (Sham) in this analysis.

Subsequently, CLP as described previously (27) was performed at 24 h post-Hem/Post-Sham Hem (Sham) on mice. In brief, following midline laparotomy, the cecum was ligated ~1 cm above the cecal tip and punctured twice with a 22G needle. Cecal contents were extruded into the intraperitoneal cavity. The abdomen was closed using a sterile PDO suture. Mice were treated with lidocaine on the muscle layer and a subcutaneous injection of 1 mL of Lactated Ringer’s solution. Mice were euthanized 24-h post procedure to isolate various tissues for downstream studies or the animal’s survival was followed for 14 days (**Supplementary Figure 3**).

### 2.4 Sample acquisition

Blood/plasma, BALFs, and lung tissues were collected as previously described at 24 h post-CLP when sequential Hem/CLP model was performed to assess Tie-2, Angiopoietin-1 (Ang-1)/Angiopoietin-2 (Ang-2), cytokine/chemokine, Evans Blue (EB) dye content (as a measurement for pulmonary vascular leak), and AST/ALT as an index of liver injury and blood urea nitrogen (BUN) as an index of kidney injury (27, 32, 33).

### 2.5 Mouse cell phenotyping by flow cytometry

Blood neutrophil (PMN, Ly6G^+^) and lung endothelial cell (EC, CD31^+^) expression of PD-L1 was analyzed from “Controls” or *ec*PD-L1**^−^**^/^**^−^** or *pmn*PD-L1**^−^**^/^**^−^** mice by flow cytometry as previously described (22, 34). Briefly, single-cell suspensions of lung tissue were prepared using a mouse lung dissociation kit (Miltenyi Biotec; cat#: 130-095-927) and gentleMACS™ dissociator as per the manufacturer’s protocol (Miltenyi Biotec, Auburn, CA) (22). Alternatively, mouse blood was processed for flow cytometric analysis as outlined in Huang et al. (34). After washing by brief centrifugation, cells were stained with various combinations of fluorochrome-conjugated antibodies as we have previously outlined (22): anti-Ly6G (clone 1A8), anti-B7-H1/PD-L1 (clone MIH5) (R&D Systems, Minneapolis, MN), and anti-CD31 (clone 390) (BD Biosciences, San Diego, CA).

Following forward and side-scatter gating, the expression of PD-L1 (**Figure 1B, C**) and the calculation %PD-L1^+^Ly6G^+^ blood PMN or %PD-L1^+^CD31^+^ EC in lung tissue (**Figure 1D, E**) were determined using a Miltenyi-MACSQuant Analyzer 10 flow cytometer (Miltenyi Biotec, Auburn, CA). To compensate for spectral overlap, UltraComp eBeads Plus Compensation Beads (Thermo Fisher Scientific: cat# 01-3333-41) were used according to the manufacturer’s protocol. Fluorescence minus one (FMO) control was used to determine positive expression gates during analysis using FlowJo software (FlowJo LLC, Ashland, OR).

### 2.6 Assessment of lung capillary leakage by extravasation of the Evans Blue dye method

Evidence of change in vascular integrity of the lungs was sought by assessing the extent of EB dye (Sigma-Aldrich: cat# E2129) extravasation (35, 36) in a separate set of animals. To assess this, mice were administered 2.5 mg of EB dye in 0.5 mL of 0.9 N saline via their tail vein. Thirty minutes later, the mice were anesthetized with isoflurane, a 0.6-mL blood plasma sample was taken, the mice were exsanguinated, and the lungs were instilled with a 2.0-mL volume of the PBS. Lung tissue specimens were weighed and minced prior to overnight extraction in 2.0 mL of formamide at 60 °C (Sigma-Aldrich: cat# 221198), after which the tissue was centrifuged at 3,000 × *g*. EB concentration in the tissue supernatant was determined spectrophotometrically at 620 nm against an EB standard curve prepared in formamide. The lung tissue content of EB is expressed as micrograms/microgram wet lung tissue.

### 2.7 Cytokine, chemokine, and angiopoietin-related protein analysis

Plasma, BALF, and lung tissue lysate samples were collected and stored as described in a previous section (27, 32, 33). To assess cytokine concentration in these samples, the following ELISA kits were used according to the manufacturer’s instructions as we have previously reported (34, 37, 38): ELISA MAX Standard Sets for mouse IL-6 (BioLegend, cat# 431301), IL-10 (BioLegend, cat# 431411), TNF-α (BioLegend, cat# 430901), and MCP-1 (BioLegend, cat# 432701); R&D Systems Duo Set Mouse ELISA kits for CXCL2/MIP-2 (R&D Systems, cat# DY452), CXCL1/KC (R&D Systems, cat# DY453), and Tie-2 (R&D Systems, cat# MTE200); MyBioSource ELISA kit for Mouse Ang-1 (MyBioSource, cat# MBS727480) as well as Abcam Mouse Ang-2 ELISA (Abcam, cat# ab171335).

### 2.8 Colorimetric assays for organ morbidity

To assess indices of tissue injury, blood was collected from mice 24 h following the sham-Hem/CLP or Hem/CLP procedure via cardiac puncture using a heparin-coated syringe. Blood sample was centrifuged at 10,000 rpm and supernatant (plasma) was collected and stored at −80 °C. For tissue injury assays, plasma was analyzed using the following kits according to the manufacturer’s protocol: Urea Nitrogen (BUN) Colorimetric Detection Kit (Invitrogen cat# EIABUN), Alanine Aminotransferase (ALT) Activity Assay Kit (Sigma-Aldrich: cat# MAK052), and Aspartate Aminotransferase (AST) Activity Assay Kit (Sigma-Aldrich: cat# MAK055).

### 2.9 Statistical analysis

Statistically significant differences between multiple groups were determined using a Kruskal–Wallis test or by a Mann–Whittney *U* test for two groups (both tests assume group data are non-parametrically distributed). Survival data were presented as a Kaplan–Meier curve, and the presence of statistically significant difference between groups was established with a log-rank survival test. Alpha was set to 0.05 as the cutoff for statistical significance using Prism 9.3.0 (GraphPad Software) statistical software. Summary data are provided as data dot-plot overlaying histograms showing the group mean and standard deviation.

## 3 Results

### 3.1 Improved survival after hemorrhagic shock and sepsis is specific to endothelial cell PD-L1 restricted gene deletion, while PD-L1 deletion in neutrophils decreases survival

**Figure 2:**
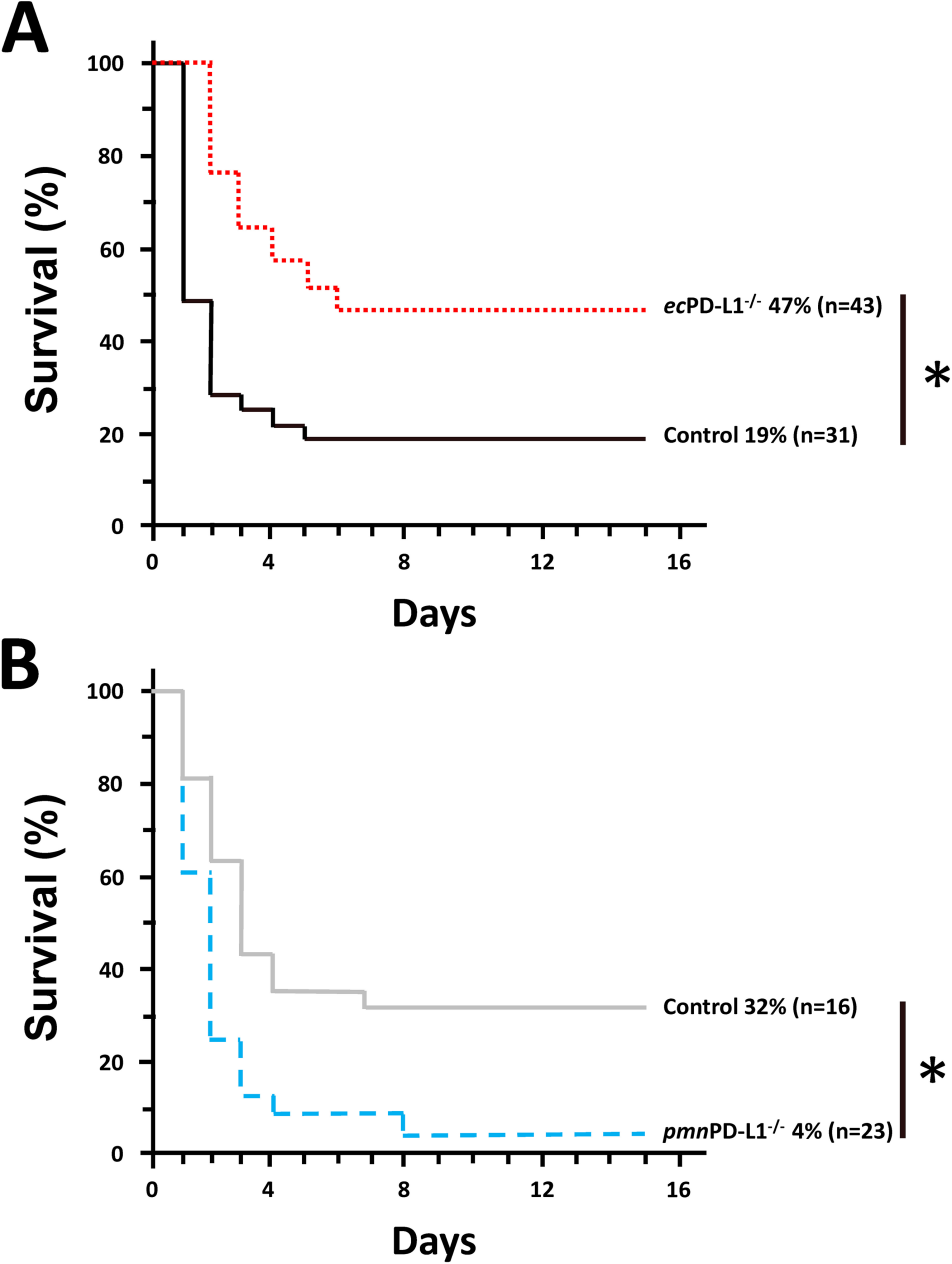
Improved survival after hemorrhagic shock and sepsis is specific to endothelial cell PD-L1 restricted gene deletion, while PD-L1 deletion in neutrophils decreases survival. *ec*PD-L1^−/−^ vs. Control **(A)** or *pmn*PD-L1^−/−^ vs. Control **(B)** mouse 14-day overall survival after subjecting them to the sequential insults of Hem/CLP as depicted by Kaplan–Meier curves. The *n*/treatment group is listed in parentheses; the presence of a significance difference between groups was established at *p < 0.05 by a log-rank test.

With respect to our initial hypothesis that endothelial cell-restricted PD-L1 gene deficiency would have protective effect on overall mortality to the sequential exposure to hemorrhagic shock followed by experimental septic challenge/CLP, we observed that the overall survival of *ec*PD-L1**^−^**^/^**^−^** mice was markedly increased over Control animals (see **Figure 2A**). Alternatively, we observed an overall decline in survival of *pmn*PD-L1**^−^**^/^**^−^** mice when compared again to their respective Control animals (see **Figure 2B**). Of note, we also looked at the septic mortality of our respective Cre-Lox Control animal groups as opposed to C57BL/6 mice obtained from The Jackson Laboratory, and while a modest rise or fall in survival can be seen between the respective Control groups when compared to the C57BL/6 animals’ response to Hem/CLP, these were not statistically significant differences in the survival of the animals over 14 days of study (**Supplementary Figure 4**). We have not included C57BL/6 mice and only utilized our in-house bred Control for all the residual studies described here relative to the *ec*PD-L1**^−^**^/^**^−^** or *pmn*PD-L1**^−^**^/^**^−^** animals.

### 3.2 With the exception of MCP-1 and IL-6, systemic blood chemokine and cytokine levels are not markedly altered by selective endothelial cell or neutrophil loss of PD-L1

**Figure 3:**
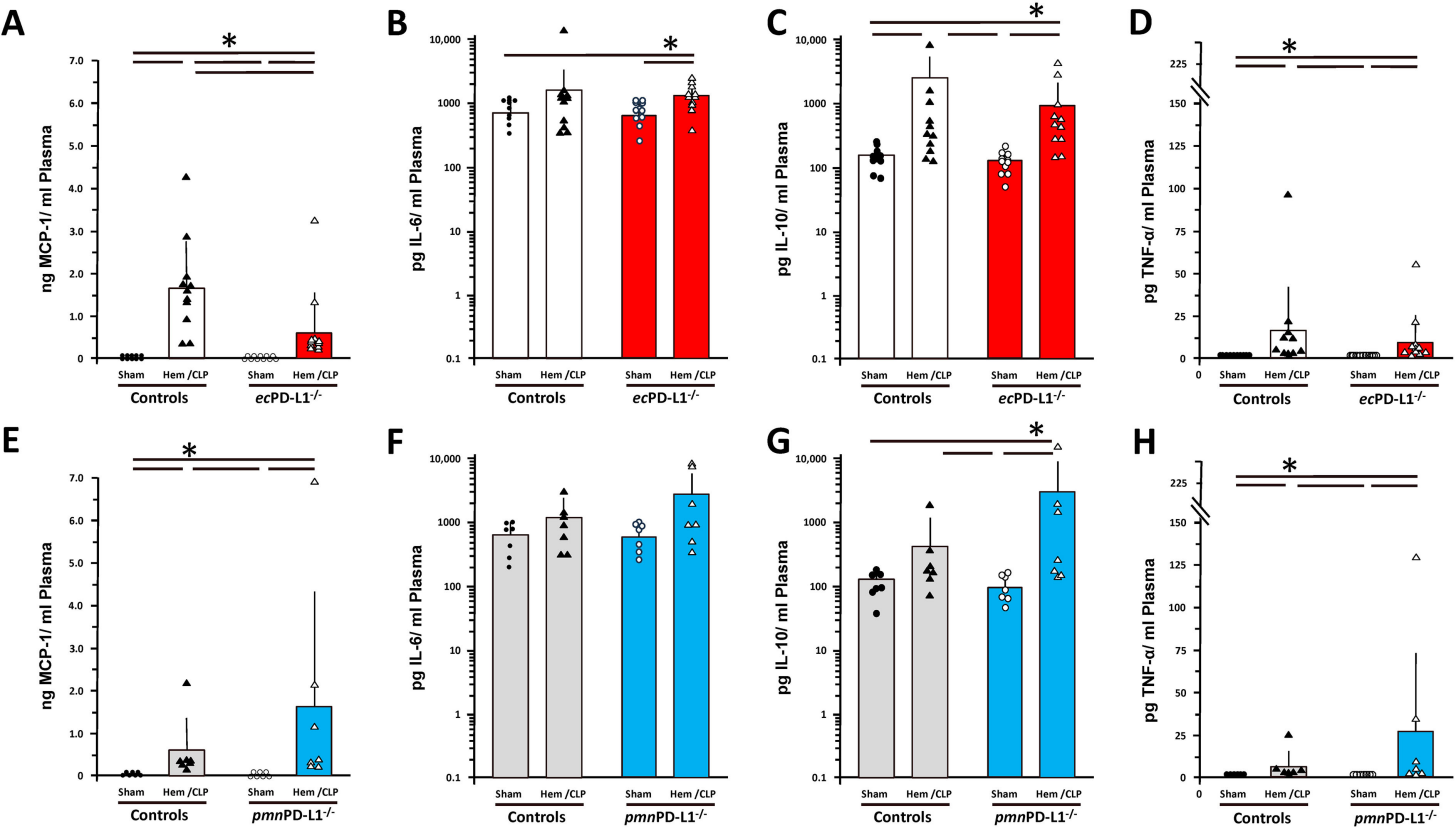
Systemic blood chemokine and cytokine levels are not markedly altered by selective endothelial cell **(A–D)** or neutrophil **(E–H)** loss of PD-L1. Systemic blood plasma chemokine/cytokine levels for MCP-1 **(A, E)**, IL-6 **(B, F)**, IL-10 **(C, G)** and TNF-α **(D, H)** were established by ELISA. The *n*/treatment group is shown as symbols super-imposed on a histogram depicting the group mean ± standard deviation; the presence of a significance difference between groups was established at *p < 0.05 by a Kruskal–Wallis multiple comparisons test.

In C57BL/6 background animals, Hem/CLP induces a marked increase in chemokines and cytokines like MCP-1, IL-10, and TNF-α, 24 h post-surgical procedure, as previously reported (30, 31) (see **Supplementary Figure 5A–D**) (see **Figures 3A, C–E, G, H**). Notably, in our models, MCP-1 levels were differentially regulated depending on whether endothelial or neutrophil PD-L1 was absent. Hem/CLP *ec*PD-L1**^−^**^/^**^−^** mice exhibited a statistically significant reduction in MCP-1 levels compared to both Sham and Hem/CLP controls (see **Figure 3A**), indicating a possible regulatory role for endothelial cell PD-L1 expression in MCP-1-mediated inflammatory responses to sepsis and shock. In contrast, *pmn*PD-L1**^−^**^/^**^−^** showed an increase in MCP-1 levels relative to the control groups; this increase, however, was not statistically significant.

Plasma IL-6 levels were increased in Hem/CLP groups compared to their Sham controls, also without statistical significance, and although plasma IL-6 was elevated in all groups post-Hem/CLP, only the Hem/CLP *ec*PD-L1**^−^**^/^**^−^** animals showed statistically significant higher levels of IL-6 than the corresponding Sham group (**Figures 3B, F**). The higher IL-6 levels in *ec*PD-L1**^−^**^/^**^−^** mice subjected to Hem/CLP, compared to their wild-type counterparts, indicate that loss of endothelial cell PD-L1 might lead to heightened inflammation by removing an important inhibitory checkpoint.

Taken together, these findings indicate that selective loss of PD-L1 on endothelial cells or neutrophils does not broadly amplify the existing systemic chemokine or cytokine responses to Hem/CLP. Instead, PD-L1 expression seems to exert cell-type-specific regulation on some inflammatory mediators: our data indicate that endothelial cell PD-L1 specifically controls the amounts of IL-6 and MCP-1, suggesting the loss of an important inhibitory checkpoint. In contrast, neutrophil-specific PD-L1 deletion had minimal impact on systemic inflammatory mediators.

### 3.3 Plasma markers of liver and kidney injury were consistently elevated after Hem/CLP, independent of cell-lineage restricted PD-L1 deletion

**Figure 4:**
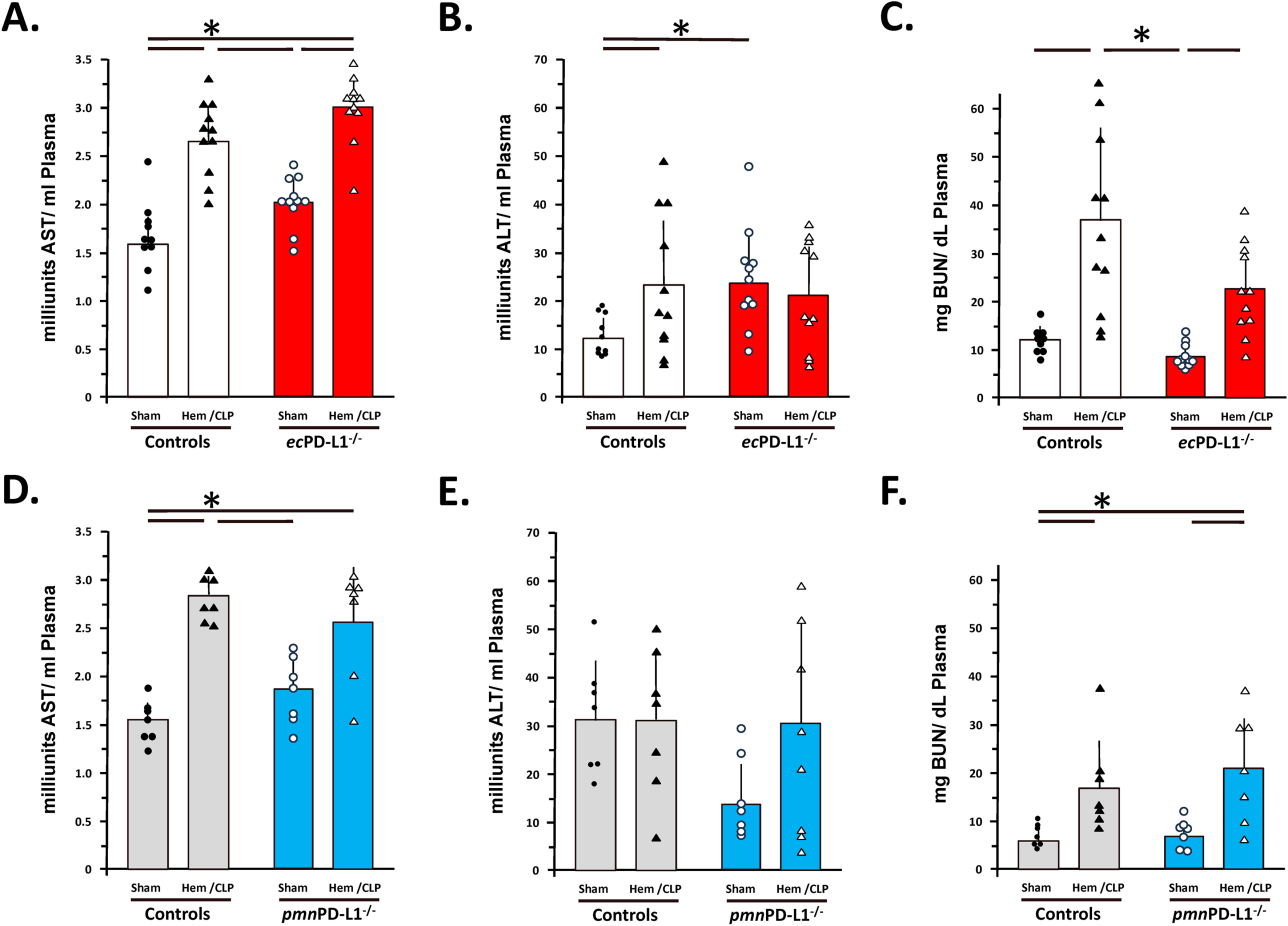
Plasma markers of liver and kidney injury were consistently elevated after Hem/CLP, independent of cell-lineage restricted PD-L1 deletion. AST **(A, D)**, ALT **(B, E)** and BUN **(C, F)** levels were established by colorimetric activity assay. The *n*/treatment group is shown as symbols super-imposed on a histogram depicting the group mean ± standard deviation; the presence of a significance difference between groups was established at *p < 0.05 by a Kruskal–Wallis multiple comparisons test.

To assess whether the differences in survival resulted from liver or kidney injury, we assessed blood levels of AST, ALT, and BUN. Even though we detected the presence of elevated levels of AST and increased BUN in all the Hem/CLP mice sera when compared to their respective Sham groups, we did not see a change in the plasma ALT levels in these same animals (**Figure 4A–F**; **Supplementary Figure 5E–G**). The differences were consistent whether the animals were wild type, *ec*PD-L1**^−^**^/^**^−^**, or *pmn*PD-L1**^−^**^/^**^−^**.

### 3.4 Endothelial cell PD-L1 gene deficiency reduces lung vascular permeability after Hem/CLP, unlike neutrophil-specific PD-L1 deletion

To determine whether the survival differences were attributable to lung injury, we first assessed lung vascular permeability after Hem/CLP using Evan’s Blue dye, a marker of pulmonary dysfunction and vascular endothelial barrier integrity.

**Figure 5:**
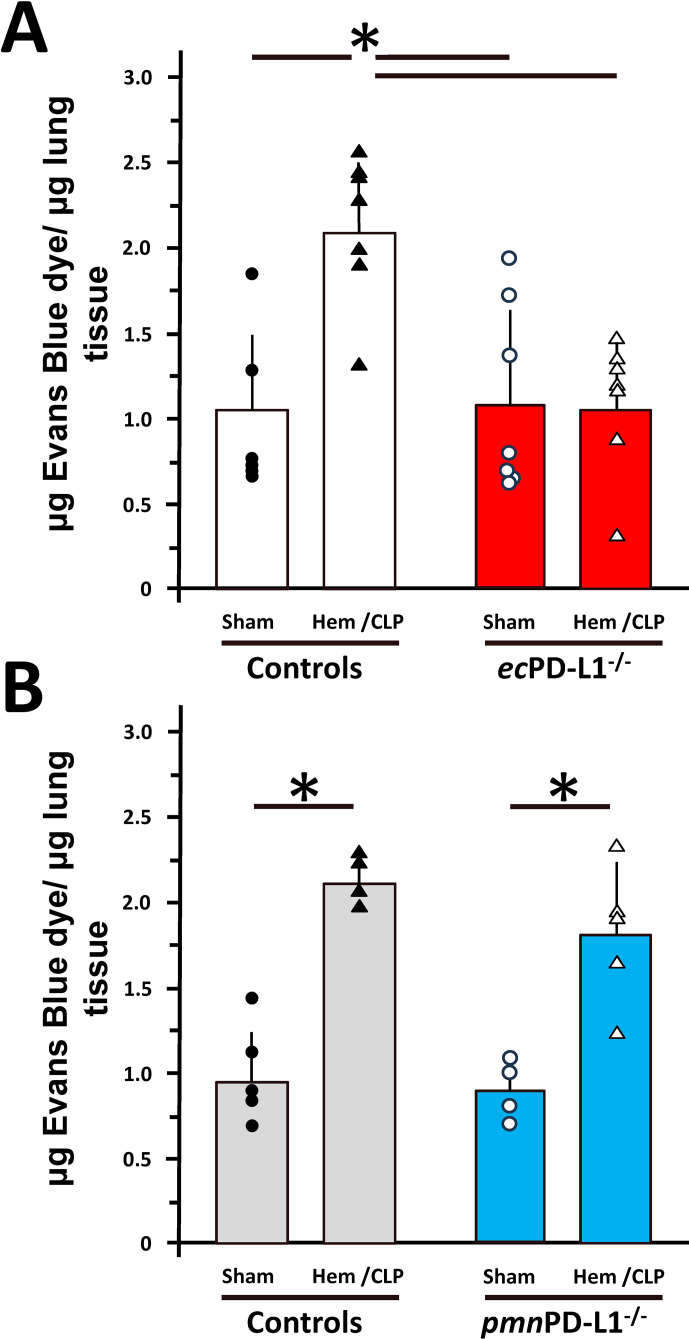
Endothelial cell PD-L1 gene deficiency **(A)** reduces lung vascular permeability after Hem/CLP, unlike neutrophil-specific PD-L1 deletion **(B)**. The assessment of lung capillary leakage was done by extravasation of EB, Evans blue dye method. The *n*/treatment group is shown as symbols super-imposed on histogram depicting the group mean ± standard deviation; the presence of a significance difference between groups was established at *p < 0.05 by a Kruskal–Wallis multiple comparisons test.

Both Control animals (**Figure 5A, B**) and C57BL/6 background mice (see **Supplementary Figure 6A**) showed a significant and consistent increase in lung permeability after Hem/CLP. Importantly, we found that the rise in lung vascular permeability was markedly attenuated in the *ec*PD-L1**^−^**^/^**^−^** Hem/CLP mice when compared to their respective Control Hem/CLP animals (see **Figure 5A**), while no such decline in permeability was seen in the lungs of *pmn*PD-L1**^−^**^/^**^−^** mice (see **Figure 5B**).

Together, these results indicate that endothelial cell PD-L1 expression plays a critical role regulating vascular lung permeability following Hem/CLP, contrary to neutrophil-specific PD-L1. Our data indicate a cell-type-specific role of PD-L1 to sepsis-induced lung injury.

### 3.5 Loss of endothelial cell PD-L1 suppresses MCP-1 in BALF, while loss of neutrophil-specific PD-L1 leads to an increase in MIP-2 and IL-6

**Figure 6:**
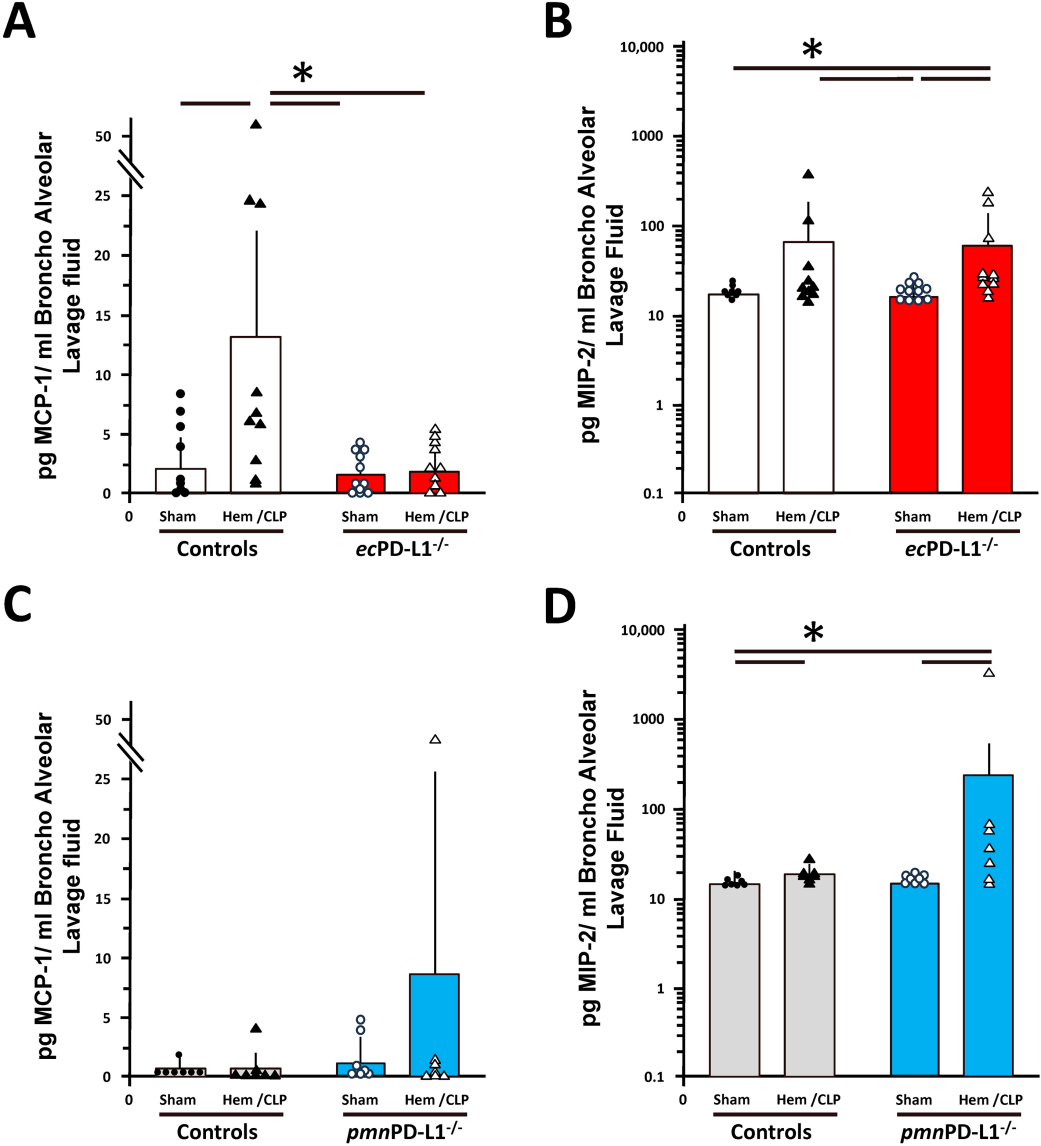
Loss of endothelial cell PD-L1 suppresses BALF MCP-1 levels **(A)**. BALF MCP-1 **(A, C)** and MIP-2 **(B, D)** chemokine levels were established by ELISA. The n/treatment group is shown as symbols super-imposed on a histogram depicting the group mean ± standard deviation; the presence of a significance difference between groups was established at *p < 0.05 by a Kruskal–Wallis multiple comparisons test.

**Figure 7:**
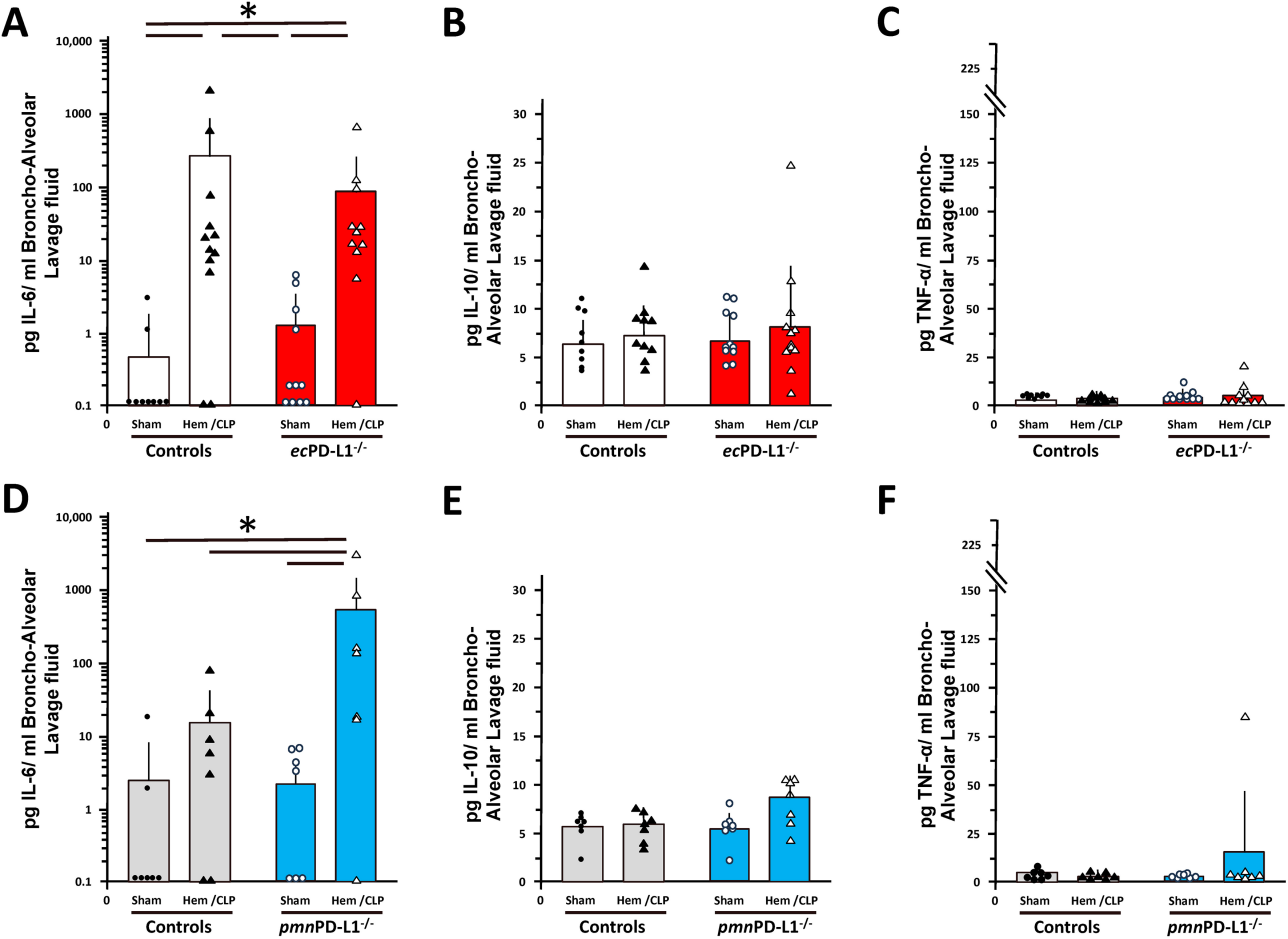
Loss of neutrophil-specific PD-L1 leads to an increase in IL-6 **(D)**. BALF IL-6 **(A, D)**, IL-10 **(B, E)** and TNF-α **(C, F)** cytokine levels were established by ELISA. The n/treatment group is shown as symbols super-imposed on a histogram depicting the group mean ± standard deviation; the presence of a significance difference between groups was established at *p < 0.05 by a Kruskal–Wallis multiple comparisons test.

As expected, subjecting Control group mice to Hem/CLP (see **Figure 6**, **7**) typically significantly increased their level of MCP-1, MIP-2, and IL-6 detected in BALF, a pattern also observed in C57BL/6 background mice (**Supplementary Figure 6B–E**). BALF levels of TNF-α and IL-10 were not changed in either *ec*PD-L1**^−^**^/^**^−^** or *pmn*PD-L1**^−^**^/^**^−^** mice subjected to Hem/CLP (see **Figures 7B, C, E, F**). Of those changes induced by Hem/CLP, the rise in MCP-1 was only attenuated in *ec*PD-L1**^−^**^/^**^−^** mice (see **Figure 6A**). Alternatively, post-Hem/CLP, BALF IL-6 levels were significantly decreased in *ec*PD-L1**^−^**^/^**^−^** mice, but significantly increased in *pmn*PD-L1**^−^**^/^**^−^** mice (**Figure 7A, D**).

### 3.6 Loss of endothelial cell PD-L1 suppresses MCP-1 levels in the lung, while loss of neutrophil-specific PD-L1 leads to an increase in IL-6

**Figure 8:**
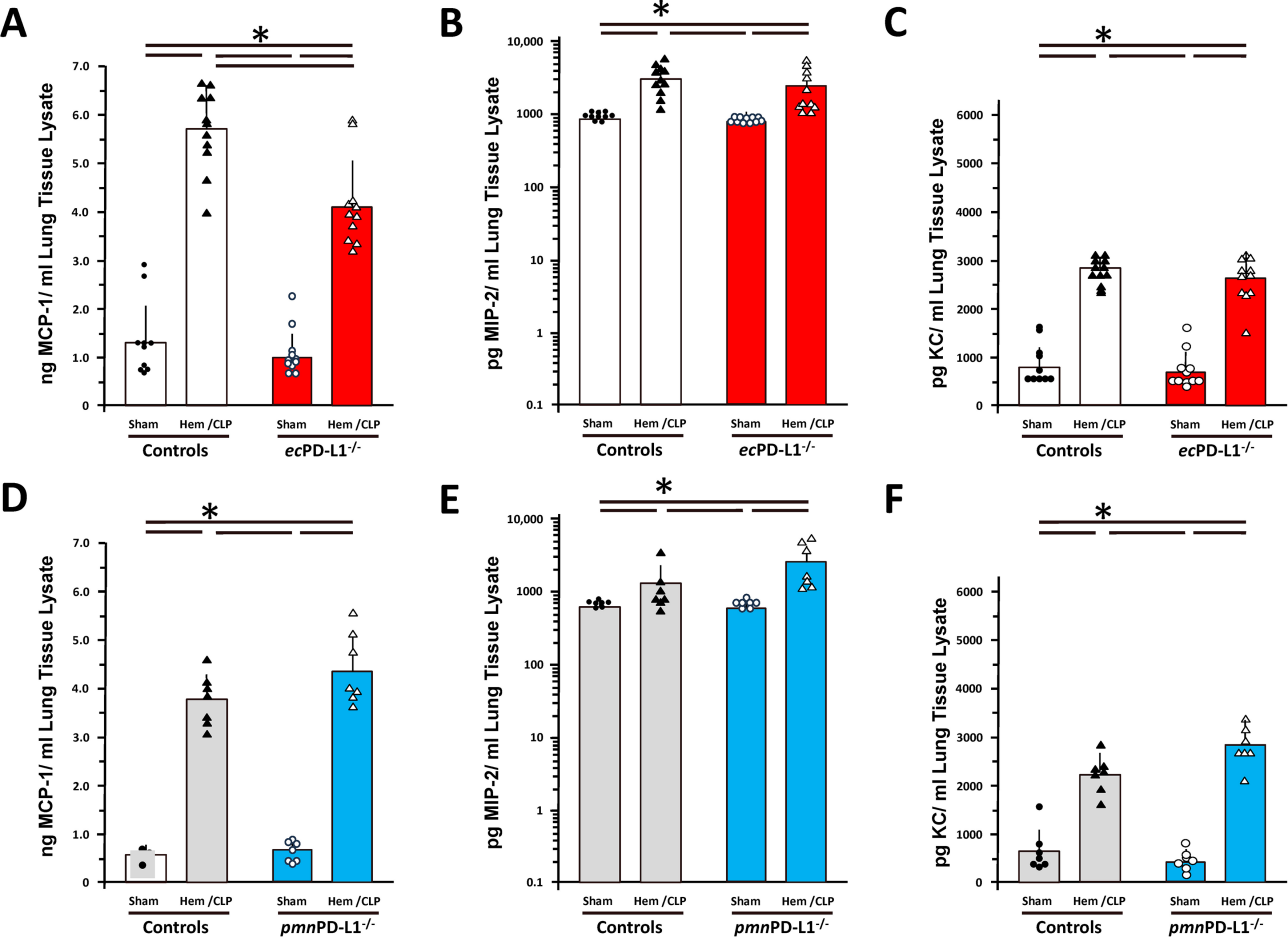
Loss of endothelial cell PD-L1 suppresses MCP-1 levels in the lungs **(A)**. Lung tissue lysate MCP-1 **(A, D)**, MIP-2 **(B, E)** and KC **(C, F)** chemokine levels were established by ELISA. The *n*/treatment group is shown as symbols super-imposed on a histogram depicting the group mean ± standard deviation; the presence of a significance difference between groups was established at *p < 0.05 by a Kruskal–Wallis multiple comparisons test.

**Figure 9:**
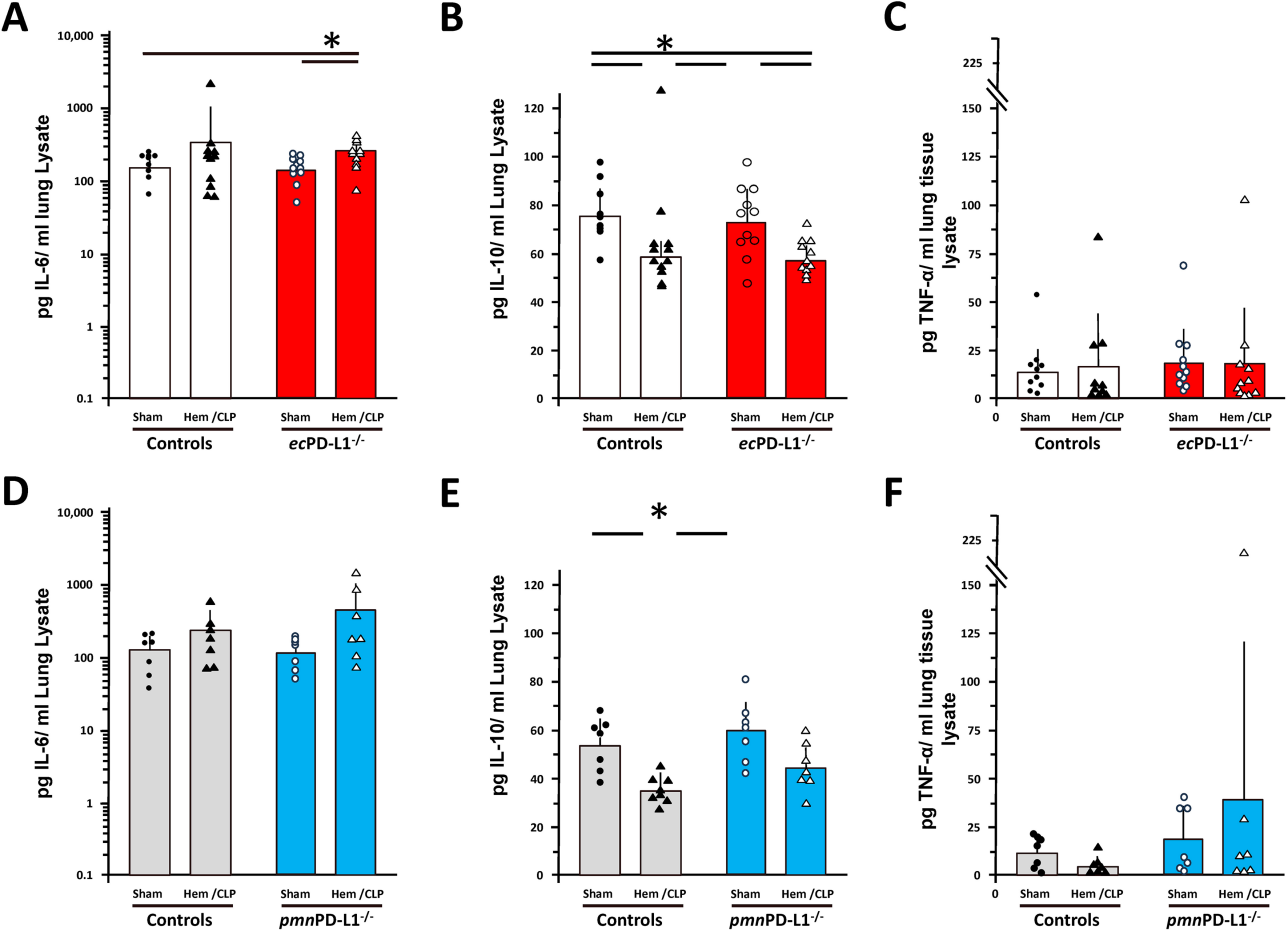
Loss of neutrophil-specific PD-L1 leads to an increase in IL-6 levels in the lungs **(D)**. Lung tissue lysate IL-6 **(A, D)**, IL-10 **(B, E)** and TNF-α **(C, F)** cytokine levels were established by ELISA. The n/treatment group is shown as symbols super-imposed on a histogram depicting the group mean ± standard deviation; the presence of a significance difference between groups was established at *p < 0.05 by a Kruskal–Wallis multiple comparisons test.

In the lung tissue itself, there was a consistent rise of MCP-1, MIP-2, and KC levels in Control mouse groups following Hem/CLP (see **Figure 8A–F**), which was comparable to the C57BL/6 background mice (see **Supplementary Figure 6F–H**). Alternatively, unlike the C57BL/6 mice, where Hem/CLP induced a marked rise in both lung tissue lysate IL-6 and TNF-α levels (see **Supplementary Figure 6I, K**), while trending higher, no statistically significant changes were observed in IL-6 as well as TNF-α in Hem/CLP *ec*PD-L1**^−^**^/^**^−^** or *pmn*PD-L1**^−^**^/^**^−^** as opposed to their respective Control animals (**Figures 9A, C, D, F**). However, there was a consistent marked decline in the Control mouse IL-10 levels following Hem/CLP of either *ec*PD-L1**^−^**^/^**^−^** or *pmn*PD-L1**^−^**^/^**^−^** (see **Figure 9B, E**), which, in one of the few discordances, were unchanged in the C57BL/6 background mice (see **Supplementary Figure 6J**).

These findings demonstrate that selective PD-L1 deletion reveals cell-specific regulation. Although Hem/CLP elevates pro-inflammatory mediators such as MCP-1, MIP-2, and IL-6, the endothelial cell-specific deletion of PD-L1 protects the lungs from increased levels of MCP-1 and IL-6 following Hem/CLP. Conversely, PD-L1 deficiency in neutrophils enhances IL-6 and MIP-2 signaling.

### 3.7 Neutrophil PD-L1 deficiency, but not endothelial cell PD-L1 loss, reduces Hem/CLP-induced changes in lung Angiopoietin 1, 2, and Tie-2 levels

**Figure 10:**
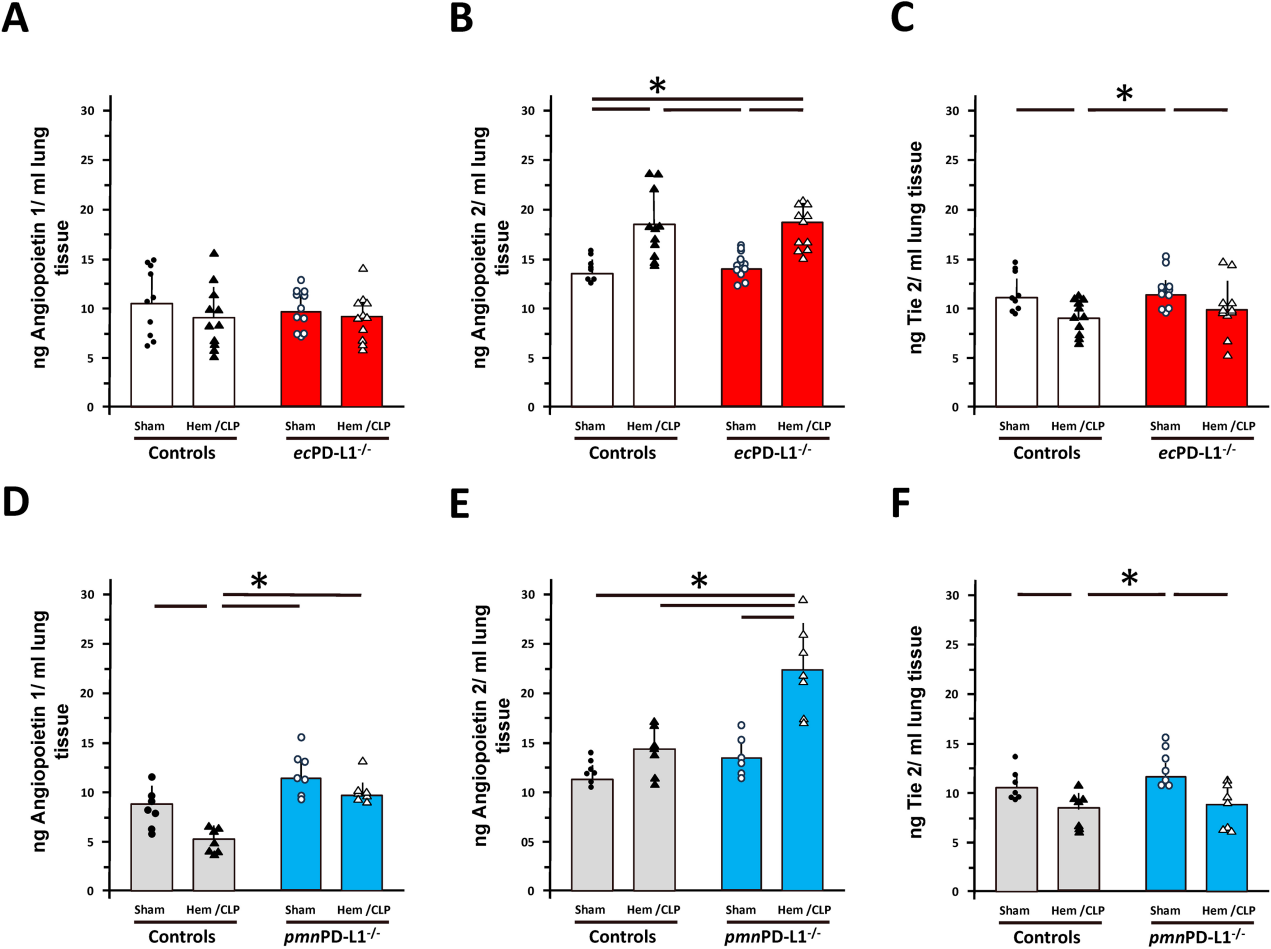
Neutrophil PD-L1 deficiency **(D–F)**, but not endothelial cell PD-L1 loss **(A–C)**, reduces Hem/CLP induced changes in lung Angiopoietin 1 **(A, D)**, 2 **(B, E)**, and Tie-2 **(C, F)** levels. Lung tissue lysate growth factor levels were established by ELISA. The n/treatment group is shown as symbols super-imposed on a histogram depicting the group mean ± the standard deviation; the presence of a significance difference between groups was established at *p < 0.05 by a Kruskal–Wallis multiple comparisons test.

Lung tissue levels of Ang-1 were unchanged in Sham vs. Hem/CLP groups in both the Control and *ec*PD-L1**^−^**^/^**^−^** mice (see **Figure 10A**), as was the case for the C57BL/6 background animals (see **Supplementary Figure 6L**). However, Ang-1 levels were markedly reduced in the Control Hem/CLP mice vs. their respective Sham group, a decline that was not observed in the Hem/CLP *pmn*PD-L1**^−^**^/^**^−^** mice when compared to their respective Sham groups (see **Figure 10D**). Twenty-four hours after Hem/CLP, there was a marked rise in lung tissue Ang-2 concentration, which was not changed in *ec*PD-L1**^−^**^/^**^−^** mice (**Figure 10B**). This was also the situation in lung tissue Ang-2 levels in Hem/CLP C57BL/6 mice (**Supplementary Figure 6M**). Alternatively, when *pmn*PD-L1**^−^**^/^**^−^** mice were subjected to Hem/CLP (**Figure 10E**), the levels of Ang-2 were markedly elevated above both the Sham and Hem/CLP Control groups, as well as the equivalent Sham *pmn*PD-L1**^−^**^/^**^−^** group. With respect to Tie-2 levels, these were significantly lower in the animals subjected to Hem/CLP (the same change was also seen for the C57BL/6 background animals; see **Supplementary Figure 6N**), and this did not change whether they were from the Control, *ec*PD-L1**^−^**^/^**^−^**, or the *pmn*PD-L1**^−^**^/^**^−^** mouse background (see **Figure 10C, F**). Taken together, these data indicate cell-specific effects of PD-L1 on pulmonary angiopoietin regulation.

## 4 Discussion

### 4.1 The results of subjecting our *ec*PD-L1^−/−^ mouse model to the sequential insults of Hem/CLP support the hypothesis we have previously proffered that increased expression of PD-L1 on endothelial cells has a morbid impact in the lungs and on overall survival

As already alluded to earlier, our laboratory and others have shown that PD-1 and its best-known ligand, PD-L1, contribute to the development of shock/sepsis**-**induced morbidity/mortality due in part to pathological interactions between myeloid cells, e.g., neutrophils, macrophages, dendritic cells, etc., or non-immune cells, such as vascular endothelial cells (22, 23, 34, 37, 39–41). However, studies utilizing global gene-deficient animal modeling do not readily permit delineation of the contribution(s) of cell-specific gene expression to the development of shock/septic organ injury (22, 23). Here, we leveraged the construction of Cre-lox mouse models by crossing an adult PD-L1^flox/flox^ mouse with either VE-Cadherin Cre or S100a8-Cre breeder to produce mice constitutively lacking either PD-L1 gene expression on endothelial cells (*ec*PD-L1**^−^**^/^**^−^**) or neutrophils (*pmn*PD-L1**^−^**^/^**^−^**), respectively. We then utilized these animals to test the hypothesis that endothelial cell, as opposed to neutrophil, deficiency of PD-L1 gene expression contributes to shock/sepsis**-**induced lung injury/death. The studies with *ec*PD-L1**^−^**^/^**^−^** mice indicated that selective loss of PD-L1 expression was associated with a marked reduction in lung vascular permeability and produced an improvement in overall survival over 14 days post-insult. This is also in keeping with the assertion we had made in a prior study by Xu et al. (23) that had compared selective differences in the cell populations that are affected by distinct routes of anti-PD-L1 siRNA delivery and/or the nature of the vehicle delivery system. In that experiment, while we were able to largely rule out pulmonary epithelial cells’ targeted sites to account for anti-PD-L1 siRNA shock/septic mouse lung protection, we could not simply say that PD-L1 gene expression induced by Hem/CLP was just due to selective targeting of vascular endothelial cells (when the anti-PD-L1 siRNA is liposomally encapsulated and given intravenously) as opposed to circulating myelocytes/leukocytes, which would also be impacted by the mode of siRNA administration. Here, the lack of such a protective effect on lung morbidity/overall mortality in the *pmn*PD-L1**^−^**^/^**^−^** as opposed to the protection seen in the *ec*PD-L1**^−^**^/^**^−^** animals supports the assertion that this is an endothelial cell-mediated effect.

**Figure 11:**
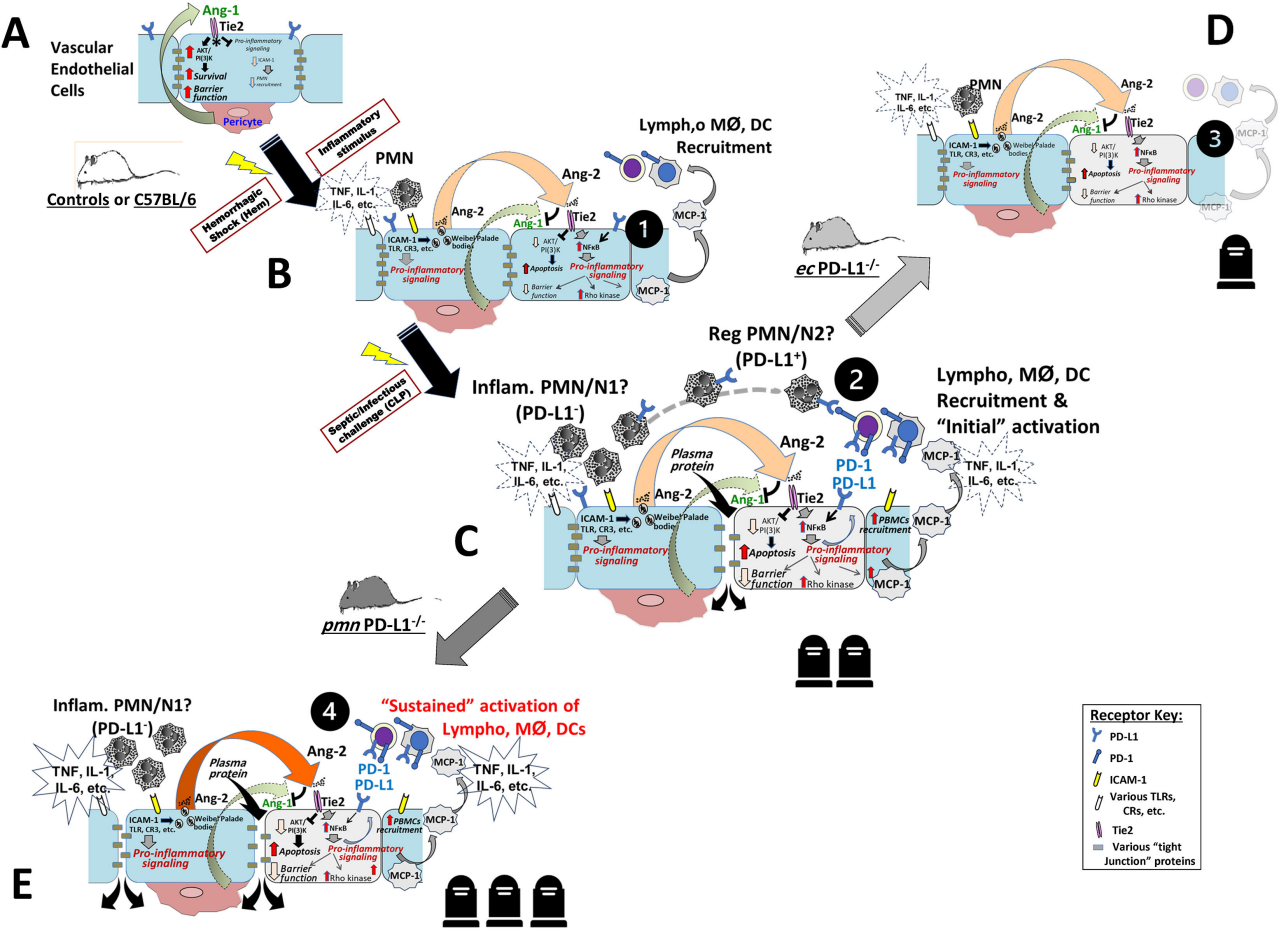
A model of how cell-restricted loss of PD-L1 expression on EC, endothelial cells or PMN, neutrophils contribute distinctly to lung morbidity and overall mortality in response to the sequential insults of Hem/CLP. Our results support the hypothesis we have previously proffered that upregulation of PD-L1 on EC, endothelial cells has a morbid impact that would seem to be due to PD-L1’s direct/indirect role (if EC PD-L1 ligated vs. no EC PD-L1 signal) in endothelial cell barrier function **(**steps **A–D)**. Somewhat surprisingly, we have also uncovered a potential immune protective role of PD-L1 expression on neutrophils, which our prior studies had not evidenced, suggesting that loss of such neutrophil expression of PD-L1 (potentially through loss of “Regulatory PMN/N2 subtype [this subtype expressed PD-L1]”; impact of PMN subtypes’ presence vs. their absence) was permissive of anti-angiogenic and select inflammatory responses that may have a role in poorer mouse survival **(**steps **A–C**, then to **E)**.

Relative to what might have accounted for this lung protective activity in the *ec*PD-L1**^−^**^/^**^−^** mice, we also examined these animals for changes in both systemic and local, BALF and lung tissue, lysate levels of pro-/anti-inflammatory chemokines/cytokines, and angiogenic factors, as well as a few select distal systemic blood indices of liver and/or kidney injury. Surprisingly, the most consistent Hem/CLP *ec*PD-L1**^−^**^/^**^−^** mouse lung tissue/BALF/blood-induced change was the suppression/attenuation of the rise in MCP-1 levels. This is interesting as it coincides with an observation we made with the global PD-L1**^−^**^/^**^−^** mice, which exhibit a marked attenuation of Hem/CLP-induced MCP-1 blood and lung tissue levels (22, 23). In this regard, MCP-1, a.k.a., CCL2, is a C-C motif chemokine family member that plays a central role in controlling macrophage, dendritic cell (but not neutrophils or eosinophils), and memory T-cell recruitment at sites of inflammation/infection (42–44). Also, while MCP-1 is produced by myeloid cells of the macrophage/monocyte lineage (45, 46), it can also be derived from vascular endothelial sources (47, 48). Thus, this makes MCP-1 an interesting potential regulatory chemokine relative to the development of EC-mediated lung injury (**Figure 11A–D**) (49, 50). That said, further experiments using either an MCP-1 neutralizing antibody or multi-gene EC-restricted knockout of PD-L1 and MCP-1 would be needed to more clearly demonstrate the significance of MCP-1 expression here.

How the loss of PD-L1 gene expression on the endothelial cells directly/indirectly acts/signals to induce changes in either endothelial cell monolayer permeability and/or mediator/chemokine release, i.e., MCP-1, is also unknown. However, precedence for such a relationship has also been seen in tumor tissue and bone marrow cells (51, 52). Beyond MCP-1, studies from our own lab by Lomas-Neira et al. (22), Xu et al. (23), Fallon et al. (53), and Wu et al. (54) applying the global PD-L1 knockout mice or selective knock-down of PD-L1 in endothelial cell lines indicated that the loss of PD-L1 gene expression appears to preserve and, in some cases, even potentiate junctional protein expression, e.g., zona occludins-1 and VE-cadherin, in the face of Hem/CLP (when endothelial cells were examined in an *ex vivo* setting) and/or experimental inflammatory challenge in the setting of tissue *in vitro* culture cell lines. Furthermore, Tao et al. (55) recently demonstrated that over-expression of PD-L1 in human lung microvascular endothelial cell cultures not only led to less junctional protein expression but also increased expression of NLRP3, cleaved-caspase-1, apoptosis-associated speck-like protein containing a CARD (a.k.a. ASC), and gasdermin D as well as potentiated mitochondrial stress. Together, this points at PD-L1-induced inflammatory pathway activation driving aspects of mitochondrial dysfunction and/or cell death (**Figures 11A–C**).

### 4.2 Alternatively, the results of subjecting our *pmn*PD-L1^−/−^ mouse model to the sequential insults of Hem/CLP did not support the hypothesis that expression of PD-L1 on neutrophils has a morbid impact in the lungs and overall survival

Interestingly, a number of studies looking at the change in expression blood leukocytes derived from patients or experimental animals have indicated that in the presence of sepsis clinically and/or in response to experimental septic challenge, there is a clear and overt rise in the expression of PD-L1 on neutrophils (56, 57). Huang et al. indicated in the setting of CLP-induced polymicrobial septic challenge that this elevated expression of PD-L1 by Ly6G^+^ (a.k.a. Gr-1^+^) blood leukocytes was associated with higher mortality (34). Wang et al. and Petra et al. both documented that increased expression of PD-L1 on circulating peripheral blood from critically ill patients with sepsis is correlated/associated with increased morbidity/mortality (56, 57). These groups both further documented that in the *ex vivo* setting, PD-L1 neutrophils derived from patients with sepsis could markedly suppress pro-inflammatory cytokine expression/secretion and exhibit restricted migratory responsiveness among other actions. This clearly implies that such cells have a potential anti-inflammatory role, which could also be seen as immune suppressive depending on the pathological setting in which it is considered. In this study, we surprisingly found that neutrophil cell lineage selective loss of PD-L1 gene expression not only was not protective against the morbidity/mortality produced in response to the sequential insults of Hem/CLP (as we saw with the *ec*PD-L1**^−^**^/^**^−^** mouse) but that it actually increased overall mortality. Considering the *ex vivo* observations of enhanced PD-L1^+^ patient neutrophil exhibiting potentiated anti-inflammatory functions, we speculate that the neutrophil-lineage selective loss of PD-L1 gene expression in our model has removed an important endogenous break on the inflammatory response to this insult (**Figure 11E**). In this respect, it is worth noting that the sequential insult of Hem/CLP markedly potentiated the *pmn*PD-L1**^−^**^/^**^−^** mouse BALF MIP-2 and IL-6 levels over the Control Hem/CLP animals. While there were trends toward increases in the *pmn*PD-L1**^−^**^/^**^−^** Hem/CLP mouse BALF: MCP-1 and IL-10; lung tissue: MCP-1, MIP-2, KC, IL-6, and TNF-α; and plasma: MCP-1, IL-6, IL-10, and TNF-α, when comparing it to *ec*PD-L1**^−^**^/^**^−^** Hem/CLP mouse levels, these were not statistically significant. However, together, they seem to speak to the loss of a potential anti-inflammatory function of the *pmn*PD-L1**^−^**^/^**^−^** mouse under these conditions. This is consistent with the observation that neutrophils that express PD-L1 are suggested to be reflective of an immune suppressive or “regulatory” and/or “N2” sub-class (58, 59) (**Figures 11C, E**). Also, as this is a “pre-dispositional”/”constitutive” neutrophil lineage selective loss of PD-L1, this would most likely have had a temporal impact on the early/acute pro-inflammatory response to these sequential insults of shock/infection.

### 4.3 Selective PD-L1 gene deficiency in *pmn*PD-L1^−/−^ led to a potentiation of the vascular growth factor Angiopoietin 2 in response to the sequential insult of shock/sepsis

Another interesting observation is that loss of PD-L1 gene expression on the neutrophil seemed to also markedly increase lung tissue Ang-2 levels. As neutrophil interaction with the endothelial cell surface has been reported to have a role in inducing the release of pre-stored mediators, of which Ang-2 is one, from the endothelial cell’s Weibel Palade bodies (32, 60, 61), it is tempting to speculate that the neutrophil expression of PD-L1 potentially serves in some way to mitigate such release (**Figures 11C, E**). Alternatively, it has been recently reported that interferon-γ-induced upregulation of endothelial cell Ang-2 gene/protein expression can also be affected indirectly by PD-L1 expression (62, 63). Thus, neutrophil expression of PD-L1 could play another anti-inflammatory role. However, whether this is a result of autocrine/paracrine actions of PD-L1 expression among neutrophils leading to the suppression of the neutrophil’s typically inflammatory activities commonly seen in response to the stimuli associated with shock/sepsis or a result of neutrophil expressed PD-L1 ligating a target on the vascular endothelial cell, possibly PD-1 (which an EC can express under inflammatory stressors, etc. (64)), remains to be established here.

### 4.4 Limitations

The most overt limitation of such an approach is that the gene deficiency created in these Cre-Lox animals are constitutive relative to the cell-lineage effect being examined; thus, it is not possible to consider the impact of gene deficiency in a temporal/post-insult fashion. When considering the significance of these cell-lineage restricted impacts of gene deficiency, we need to appreciate the pre-dispositional nature of such a loss on the pathology here as opposed to a more temporally specific treatment-like pharmacological or antibody inhibition. In this regard, it is worth noting that while less cell-lineage selective in nature, Xu et al.’s (23) use of liposomally encapsulated PD-L1 siRNA intravenous delivery at 2 h post-Hem/CLP to mitigate morbid effects of this dual-insult indirect ARDS model supports PD-L1 as a potential early (2–24 h post-insult) temporal target. Also, as Xu et al. (23) observed a reduction in lung morbidity and several systemic plasma pro-inflammatory cytokines/chemokines (including the chemokine MCP-1 that we have reported was effected in this study) under these delivery conditions, it suggests that the predominant pathological activity was likely also at the vascular endothelial cell interface and not so much due to its actions of PD-L1 expressing neutrophils. Nonetheless, as with all models, care needs to be taken when interpreting the translational potential of such constitutive Cre-Lox mouse studies.

Another limitation of this work is that it was done utilizing young, mature 8- to 12-week-old male mice in an attempt to maximize the chance of seeing differences in the response to sequential insults of shock/sepsis for the reasons of more morbid immune/organ responsiveness we eluded to in the Materials and methods section (28–31). Thus, studies are still needed in both equivalent aged estrous-stratified or non-stratified females (30), which would express the differing impacts of biological aspects of sex, as well as the effects of age (65, 66) and other potential comorbidities, and impact the endothelial cell and neutrophil-driven responses seen here in the lung ALI.

### 4.5 Conclusions

Taken together, these study results we have presented here support the hypothesis we have previously proffered, that expression of PD-L1, specifically when upregulated on endothelial cells, has a morbid impact that would seem to be due to PD-L1’s direct/indirect role in endothelial cell barrier function possibly mediated by MCP-1/CCL2 activity. Surprisingly, we have also uncovered a potential immune protective role of PD-L1 expression on neutrophils, which our prior studies had not evidenced, suggesting that loss of such myeloid cell expression of PD-L1 permitted anti-angiogenic and select inflammatory responses that may have a role in poorer mouse survival (**Figure 11**). It is tempting to speculate that such divergent morbid activities of cell-specific PD-L1 expression might potentially explain the mixed results seen in clinical trials targeting the PD-1/PD-L1 axis in patients with sepsis (67–70).

## Data Availability

The datasets analyzed for this study can be found in the Brown University and within the Supplement section of this article: https://sites.brown.edu/ayala-lab/files/2026/02/AA_Data-for-CreLox-PDL1-flox-mice-study-2.2026.xlsx.

## References

[1] Tsukamoto T Chanthaphavong RS Pape HC . Current theories on the pathophysiology of multiple organ failure after trauma. Injury. (2010) 41:21–6. doi: 10.1016/j.injury.2009.07.010. PMID: 19729158

[2] Yao YM Redl H Bahrami S Schlag G . The inflammatory basis of trauma/shock-associated multiple organ failure. Inflammation Res. (1998) 47:210. doi: 10.1007/s000110050318. PMID: 9657252

[3] Regel G Grotz M Weltner T Sturm JA Tscherne H . Pattern of organ failure following severe trauma. World J Surg. (1996) 20:422–9. doi: 10.1007/s002689900067. PMID: 8662130

[4] Choi J Carlos G Nassar AK Knowlton LM Spain DA . The impact of trauma systems on patient outcomes. Curr Probl Surg. (2021) 58:100849. doi: 10.1016/j.cpsurg.2020.100840. PMID: 33431134 PMC7286246

[5] Sun GD Zhang Y Mo SS Zhao MY . Multiple organ dysfunction syndrome caused by sepsis: Risk factor analysis. Int J Gen Med. (2021) 14:7159–64. doi: 10.2147/ijgm.s328419. PMID: 34737610 PMC8559339

[6] Zhang N Liu YJ Yang C Zeng P Gong T Tao L . Review of research progress on the role of the effective components of traditional Chinese medicine in sepsis with multiple organ dysfunction. Heliyon. (2023) 9:e21713. doi: 10.1016/j.heliyon.2023.e21713. PMID: 38027612 PMC10665755

[7] Singer M Deutschland CS Seymour CW Shankar-Hari M Annan D Bauer M . The third international consensus definitions for sepsis and septic shock (sepsis-3). JAMA. (2016) 315:801–10. doi: 10.1001/jama.2016.0287. PMID: 26903338 PMC4968574

[8] Xu J Kochanek KD Murphy SL Tejada-Vera B . Deaths: final data for 2007. Natl Vital Stat Rep (CDC). (2010) 58:1–135. doi: 10.15620/cdc:131355 25075874

[9] Rittirsch D Flierl MA Ward PA . Harmful molecular mechanisms in sepsis. Nat Rev Immunol. (2008) 8:776–87. doi: 10.1038/nri2402. PMID: 18802444 PMC2786961

[10] U.S. Food & Drug Administration . FDA drug safety communication (10/25/2011): Voluntary market withdrawal of Xigris [drotrecogin alfa (activated)] due to failure to show survival benefit (2011). Available online at: https://www.fda.gov/drugs/drug-safety-and-availability/fda-drug-safety-communication-voluntary-market-withdrawal-xigris-drotrecogin-alfa-activated-due (Accessed June 24, 2012).

[11] Matsuyama K . Eisai's sepsis drug Eritoran fails to save more lives in final-stage study (2011). Available online at: http://www.bloomberg.com/news/2011-01-25/eisai-s-sepsis-drug-eritoran-fails-to-save-more-lives-in-final-stage-study.html (Accessed June 24, 2012).

[12] Zompatori M Ciccarese F Fasano L . Overview of current lung imaging in acute respiratory distress syndrome. Eur Respir Rev. (2014) 23:519–30. doi: 10.1183/09059180.00001314. PMID: 25445951 PMC9487404

[13] Matthay MA Zimmerman GA . Acute lung injury and the acute respiratory distress syndrome: four decades of inquiry into pathogenesis and rational management. Amer J Respir Cell Mol Biol. (2005) 33:319–27. doi: 10.1146/annurev-pathol-011110-130158. PMID: 16172252 PMC2715340

[14] Parsey MV Tuder RM Abraham E . Neutrophils are major contributors to intraparenchymal lung IL-1b expression after hemorrhage and endotoxemia. J Immunol. (1998) 160:1007–13. doi: 10.4049/jimmunol.160.2.1007 9551941

[15] Shenkar R Abraham E . Mechanisms of neutrophil activation after hemorrhage or endotoxemia: roles of reactive oxygen intermediates, NF-kappa B and cyclic AMP response element binding protein. J Immunol. (1999) 163:954–62. doi: 10.4049/jimmunol.163.2.954 10395692

[16] Doerschuk CM . Mechanisms of leukocyte sequestration in inflamed lungs. Microcirculation. (2001) 8:71–88. doi: 10.1111/j.1549-8719.2001.tb00159.x. PMID: 11379793

[17] Perl M Lomas-Neira J Chung CS Ayala A . Epithelial cell apoptosis and neutrophil recruitment in acute lung injury-a unifying hypothesis? What we have learned from small interfering RNAs. Mol Med. (2008) 14:465–75. doi: 10.2119/2008-00011.perl. PMID: 18368145 PMC2274893

[18] Zimmerman GA Albertine KH Carveth HJ Gill EA Grissom CK Hoidal JR . Endothelial activation in ARDS. Chest. (1999) 116:18s–24s. doi: 10.1378/chest.116.suppl_1.18s. PMID: 10424566

[19] Ware LB Matthay MA . Alveolar fluid clearance is impaired in the majority of patients with acute lung injury and the acute respiratory distress syndrome. Amer J Respir Crit Care Med. (2001) 163:1376–83. doi: 10.1164/ajrccm.163.6.2004035. PMID: 11371404

[20] Perl M Chung CS Perl U Lomas-Neira JL De Paepe ME Cioffi WG . Fas induced pulmonary apoptosis and inflammation during indirect acute lung injury. Amer J Respir Crit Care Med. (2007) 176:591–601. doi: 10.1164/rccm.200611-1743oc. PMID: 17600273 PMC1994224

[21] Ayala A . Mechanisms of immune suppression in sepsis/shock: One investigator's/lab group's experience (SLB 2024 Legacy Award Presentation). J Leukoc Biol. (2025) 117:qiaf108. doi: 10.1093/jleuko/qiaf108. PMID: 40702670 PMC12366726

[22] Lomas-Neira JL Monaghan SF Huang X Fallon EA Chung CS Ayala A . Novel role for PD-1:PD-L1 as a mediator of pulmonary vascular endothelial cell functions in the pathogenesis of indirect ARDS in mice. Front Immunol. (2018) 9:3030. doi: 10.3389/fimmu.2018.03030. PMID: 30619369 PMC6306416

[23] Xu S Yang Q Bai J Tao T Tang L Chen Y . Blockade of endothelial, but not epithelial, cell expression of PD-L1 following severe shock attenuates the development of indirect acut lung injury in mice. Amer J Physiol. (2020) 318:L801–12. doi: 10.1152/ajplung.00108.2019. PMID: 31994912 PMC7191484

[24] Gray CC Biron-Girard B Wakeley ME Chung CS Chen Y Quiles-Ramirez Y . Negative immune checkpoint protein, VISTA, regulates the CD4(+) T(reg) population during sepsis progression to promote acute sepsis recovery and survival. Front Immunol. (2022) 13:861670. doi: 10.3389/fimmu.2022.861670. PMID: 35401514 PMC8988198

[25] Monach PA Mathis D Benoist C . The K/BxN arthritis model. Curr Protoc Immunol. (2008) Chapter 15:15.22.1–15.22.12. doi: 10.1002/0471142735.im1522s81. PMID: 18491295

[26] Latchman YE Liang SC Wu Y Chernova T Sobel RA Klemm M . PD-L1-deficient mice show that PD-L1 on T cells, antigen-presenting cells, and host tissues negatively regulates T cells. Proc Natl Acad Sci USA. (2004) 101:10691–6. doi: 10.1073/pnas.0307252101. PMID: 15249675 PMC489996

[27] Ayala A Chung CS Lomas JL Song GY Doughty LA Gregory SH . Shock induced neutrophil mediated priming for acute lung injury in mice: divergent effects of TLR-4 and TLR-4/FasL deficiency. Amer J Pathol. (2002) 161:2283–94. doi: 10.1016/S0002-9440(10)64504-X PMC1850899 12466142

[28] Wichmann MW Ayala A Chaudry IH . Male sex steroids are responsible for depressing macrophage immune function after trauma-hemorrhage. Amer J Physiol. (1997) 273:C1335–1340. doi: 10.1152/ajpcell.1997.273.4.c1335. PMID: 9357778

[29] Zellweger R Wichmann MW Ayala A Stein S DeMaso CM Chaudry IH . Females in proestrus state maintain splenic immune functions and tolerate sepsis better than males. Crit Care Med. (1997) 25:106–10. doi: 10.1097/00003246-199701000-00021. PMID: 8989185

[30] Schneider CP Schwacha MG Chaudry IH . Impact of sex and age on bone marrow immune responses in a murine model of trauma-hemorrhage. J Appl Physiol. (2007) 102:113–21. doi: 10.1152/japplphysiol.00848.2006. PMID: 17023570

[31] Wang M Crisostomo P Wairiuko GM Meldrum DR . Estrogen receptor-alpha mediates acute myocardial protection in females. Amer J Physiol. (2006) 290:H2204–9. doi: 10.1152/ajpheart.01219.2005. PMID: 16415070

[32] Lomas-Neira J Venet F Chung C-S Thakkar RK Heffernan DS Ayala A . Neutrophil-endothelial interactions mediate angiopoietin-2 associated pulmonary cell dysfunction in idirect ALI in mice. Amer J Respir Cell Mol Biol. (2014) 50:193–200. doi: 10.1165/rcmb.2013-0148OC 23980650 PMC3930935

[33] Lomas-Neira JL Perl M Venet F Chung CS Ayala A . The role and source of TNF-alpha in hemorrhage induced priming for septic lung injury. Shock. (2012) 37:611–20. doi: 10.1097/SHK.0b013e318254fa6a PMC3356491 22552013

[34] Huang X Chen Y Chung C-S Yuan Z Monaghan SF Wang F . Identification of B7-H1 as a novel mediator of the innate immune/proinflammatory response a well as a possible myeloid cell prognostic biomarker in sepsis. J Immunol. (2014) 192:1091–9. doi: 10.4049/jimmunol.1302252. PMID: 24379123 PMC3947010

[35] Moxley MA Baird TL Corbett JA . Adoptive transfer of acute lung injury. Amer J Physiol. (2000) 279:L985–93. doi: 10.1152/ajplung.2000.279.5.l985. PMID: 11053036

[36] Lomas-Neira J Heffernan DS A. A Monaghan SF . Blockade of endothelial growth factor, Angiopietin-2, reduces indices of ARDS and mortality in mice resulting from the dual-insults of hemorrhagic shock and sepsis. Shock. (2016) 45:157. doi: 10.1097/shk.0000000000000499. PMID: 26529660 PMC4715730

[37] Huang X Venet F Wang YL Lepape A Yuan Z Chen Y . PD-1 expression by macrophages plays a pathologic role in altering microbial clearance and the innate inflammatory response to sepsis. Proc Natl Acad Sci USA. (2009) 106:6303–8. doi: 10.1073/pnas.0809422106. PMID: 19332785 PMC2669369

[38] Zhu J Li J Chung CS Lomas-Neira JL Ayala A . Patho-mechanisms for hemorrhage/sepsis-induced indirect acute respiratory distress syndrome: A role for lung TIE1 and its regulation by neutrophils. Shock. (2022) 57:608–15. doi: 10.1097/shk.0000000000001902. PMID: 34907117 PMC8916968

[39] Monaghan SF Thakkar RK Heffernan DS Tran HL Huang X Chung CS . Mechanisms of indirect acute lung injury: a novel role for the co-inhibitory receptor, programmed death-1 (PD-1). Ann Surg. (2012) 255:158–64. doi: 10.1097/sla.0b013e31823433ca. PMID: 21997806 PMC3243770

[40] Brahmamdam P Inoue S Unsinger J Chang KC McDunn JE Hotchkiss RS . Blocking PD-1 inhibits lymphocyte apoptosis during sepsis. J Leukoc Biol. (2010) 88:233–40. doi: 10.1189/jlb.0110037 PMC6607999 20483923

[41] Boomer JS To K Chang KC Takasu O Osborne DF Walton AH . Immunosuppression in patients who die of sepsis and multiple organ failure. JAMA. (2011) 306:2594–605. doi: 10.1097/sa.0b013e3182751ec1. PMID: 22187279 PMC3361243

[42] Evers TMJ Sheikhhassani V Haks MC Storm C Ottenhoff THM Mashaghi A . Single-cell analysis reveals chemokine-mediated differential regulation of monocyte mechanics. iScience. (2022) 25:103555. doi: 10.1016/j.isci.2021.103555. PMID: 34988399 PMC8693412

[43] Carr MW Roth SJ Luther E Rose SS Springer TA . Monocyte chemoattractant protein 1 acts as a T-lymphocyte chemoattractant. Proc Natl Acad Sci USA. (1994) 91:3652–6. doi: 10.1073/pnas.91.9.3652. PMID: 8170963 PMC43639

[44] Xu LL Warren MK Rose WL Gong W Wang JM . Human recombinant monocyte chemotactic protein and other C-C chemokines bind and induce directional migration of dendritic cells *in vitro*. J Leukoc Biol. (1996) 60:365–71. doi: 10.1002/jlb.60.3.365. PMID: 8830793

[45] Yoshimura T Yuhki N Moore SK Appella E Lerman MI Leonard EJ . Human monocyte chemoattractant protein-1 (MCP-1). Full-length cDNA cloning, expression in mitogen-stimulated blood mononuclear leukocytes, and sequence similarity to mouse competence gene JE. FEBS Lett. (1989) 244:487–93. doi: 10.1016/0014-5793(89)80590-3. PMID: 2465924

[46] Yoshimura T . The chemokine MCP-1 (CCL2) in the host interaction with cancer: a foe or ally? Cell Mol Immunol. (2018) 15:335–45. doi: 10.1038/cmi.2017.135. PMID: 29375123 PMC6052833

[47] Khyzha N Khor M DiStefano PV Wang L Matic L Hedin U . Regulation of CCL2 expression in human vascular endothelial cells by a neighboring divergently transcribed long noncoding RNA. Proc Natl Acad Sci USA. (2019) 116:16410–9. doi: 10.1016/j.atherosclerosissup.2018.04.288. PMID: 31350345 PMC6697820

[48] Thomas D Noishiki C Gaddam S Wu D Manhas A Liu Y . CCL2-mediated endothelial injury drives cardiac dysfunction in long COVID. Nat Cardiovasc Res. (2024) 3:1249–65. doi: 10.1038/s44161-024-00543-8. PMID: 39402206 PMC12243935

[49] Ziraldo C Vodovotz Y Namas RA Almahmoud K Tapias V Mi Q . Central role for MCP-1/CCL2 in injury-induced inflammation revealed by *in vitro*, in silico, and clinical studies. PloS One. (2013) 8:e79804. doi: 10.1016/j.jcrc.2013.07.005. PMID: 24312451 PMC3849193

[50] Azhar N Namas RA Almahmoud K Zaaqoq A Malak OA Barclay D . A putative "chemokine switch" that regulates systemic acute inflammation in humans. Sci Rep. (2021) 11:9703. doi: 10.1038/s41598-021-88936-8 33958628 PMC8102583

[51] Wellhausen J Röhl L Berszin M Krücken I Zebralla V Pirlich M . Suppression of MCP-1, IFN-γ and IL-6 production of HNSCC ex vivo by pembrolizumab added to docetaxel and cisplatin (TP) exceeding those of TP alone is linked to improved survival. Front Immunol. (2024) 15:1473897. doi: 10.3389/fimmu.2024.1473897. PMID: 39882242 PMC11774711

[52] Costa SN Chlebek C Gray L Caradonna P Ryzhov S Rosen CJ . Expansion of bone marrow adipocytes in obese mice leads to PD-L1-driven bone marrow immunosuppression and osteoclastogenesis. Bone Res. (2026) 14:32. doi: 10.1038/s41413-026-00509-5. PMID: 41856986 PMC13002983

[53] Fallon EA Chung CS Heffernan DS Chen Y De Paepe ME Ayala A . Survival and pulmonary injury after neonatal sepsis: PD1/PDL1's contributions to mouse and human immunopathology. Front Immunol. (2021) 12:634529. doi: 10.3389/fimmu.2021.634529. PMID: 33746973 PMC7965961

[54] Wu Y Chung CS Chen Y Monaghan SF Patel S Huang X . A novel role for programmed cell death receptor ligand-1 in sepsis-induced intestinal dysfunction. Mol Med. (2016) 22:830–40. doi: 10.2119/molmed.2016.00150. PMID: 27782294 PMC5263053

[55] Tao T Zhu Y Shi Y Sun B Gu Y Xu S . Unveiling the role of PD-L1 in vascular endothelial dysfunction: Insights into the mtros/NLRP3/caspase-1 mediated pyroptotic pathway. Exp Cell Res. (2024) 438:114047. doi: 10.1016/j.yexcr.2024.114047. PMID: 38631546

[56] Patera AC Drewry AM Chang K Beiter ER Osborne D Hotchkiss RS . Frontline science: Defects in immune function in patients with sepsis are associated with PD-1 or PD-L1 expression and can be restored by antibodies targeting PD-1 or PD-L1. J Leukoc Biol. (2016) 100:1239–54. doi: 10.1189/jlb.4hi0616-255r. PMID: 27671246 PMC5110001

[57] Wang JF Li JB Zhao YJ Yi WJ Bian JJ Wan XJ . Upregulation of programmed cell death 1 ligand 1 on neutrophils may be involved in sepsis-induced immunosuppression: An animal study and a prospective case-control study. Anesthesiology. (2015) 122:852–63. doi: 10.1097/ALN.0000000000000525 25437496

[58] Ganesh K Joshi MB . Neutrophil sub-types in maintaining immune homeostasis during steady state, infections and sterile inflammation. Inflammation Res. (2023) 72:1175–92. doi: 10.1007/s00011-023-01737-9. PMID: 37212866 PMC10201050

[59] Vanhaver C Aboubakar Nana F Delhez N Luyckx M Hirsch T Bayard A . Immunosuppressive low-density neutrophils in the blood of cancer patients display a mature phenotype. Life Sci Alliance. (2024) 7:e202302332. doi: 10.26508/lsa.202302332. PMID: 37931958 PMC10628041

[60] Fiedler U Scharpfenecker M Koidl S Hegen A Grunow V Schmidt JM . The tie-2 ligand angiopoietin-2 is stored in and rapidly released upon stimulation from endothelial cell Weibel-Palade bodies. Blood. (2004) 103:4150–6. doi: 10.1182/blood-2003-10-3685. PMID: 14976056

[61] Fiedler U Reiss Y Scharpfenecker M Grunow V Koidl S Thurston G . Angiopoietin-2 sensitizes endothelial cells to TNF-a and has a crucial role in the induction of inflammation. Nat Med. (2006) 12:235–9. doi: 10.1038/nm1351. PMID: 16462802

[62] Cai Z Meng K Yu T Xi Y Yuan Z Wang X . IFN-γ-mediated suppression of ANGPT2-Tie2 in endothelial cells facilitates tumor vascular normalization during immunotherapy. Front Immunol. (2025) 16:1551322. doi: 10.3389/fimmu.2025.1551322. PMID: 40370455 PMC12075545

[63] Gao Y Yang J Cai Y Fu S Zhang N Fu X . IFN-γ-mediated inhibition of lung cancer correlates with PD-L1 expression and is regulated by PI3K-AKT signaling. Int J Cancer. (2018) 143:931–43. doi: 10.1002/ijc.31357. PMID: 29516506

[64] He T Zhang M Qin J Wang Y Li S Du C . Endothelial PD-1 regulates vascular homeostasis and oligodendrogenesis during brain development. Adv Sci (Weinh). (2025) 12:e2417410. doi: 10.6019/s-biad1416 40013943 PMC12021089

[65] Nomellini V Gomez CR Kovacs EJ . Aging and impairment of innate immunity. Contrib Microbiol. (2008) 15:188–205. doi: 10.1159/000136358. PMID: 18511862

[66] Kovacs EJ Boe DM Boule LA Curtis BJ . Inflammaging and the lung. Clin Geriatr Med. (2017) 33:459–71. doi: 10.1016/j.cger.2017.06.002. PMID: 28991644 PMC5653264

[67] Hotchkiss RS Colston E Yende S Angus DC Moldawer LL Crouser ED . Immune checkpoint inhibition in sepsis: A phase 1b randomized, placebo-controlled, single ascending dose study of antiprogrammed cell death-ligand (BMS-936559). Crit Care Med. (2019) 45:1360–71. doi: 10.1097/ccm.0000000000003685. PMID: 30747773 PMC7254685

[68] Hotchkiss RS Colston E Yende S Crouser ED Martin GS Albertson T . Immune checkpoint inhibition in sepsis: A phase 1b randomized study to evaluate the safety, tolerability, pharmacokinetics, and pharmacodynamics of nivolumab. Intensive Care Med. (2019) 45:1360–71. doi: 10.1007/s00134-019-05704-z. PMID: 31576433 PMC9006384

[69] Watanabe E Nishida O Kakihana Y Odani M Okamura T Harada T . Pharmacokinetics, pharmacodynamics, and safety of nivolumab in patients with sepsis-induced immunosuppression: A multicenter, open-label phase 1/2 study. Shock. (2020) 53:686–94. doi: 10.1097/shk.0000000000001443. PMID: 31513050 PMC7448837

[70] Rienzo M Skirecki T Monneret G Timsit JF . Immune checkpoint inhibitors for the treatment of sepsis: Insights from preclinical and clinical development. Expert Opin Investig Drugs. (2022) 31:885–94. doi: 10.1080/13543784.2022.2102477. PMID: 35944174

