# Supplementary material for: Endothelial cell, but not neutrophil, programmed cell death receptor-ligand 1 loss has a morbid impact on experimental murine shock/sepsis-induced lung injury
Source: Front Immunol. 2026 Jun 2;17:1816915. doi: 10.3389/fimmu.2026.1816915 (PMC13268903; doi:10.3389/fimmu.2026.1816915)

Supplemental Figure 2. Typical neutrophil restricted PD-L1 deficient (*pmn*PD-L1$^{-/-}$) and 'Control' breeding mice construction process based on crosses of PD-L1$^{flox/flox}$ animals with B6.Cg-Tg(S100a8-cre,-EGFP)1Ilw/J breeders.

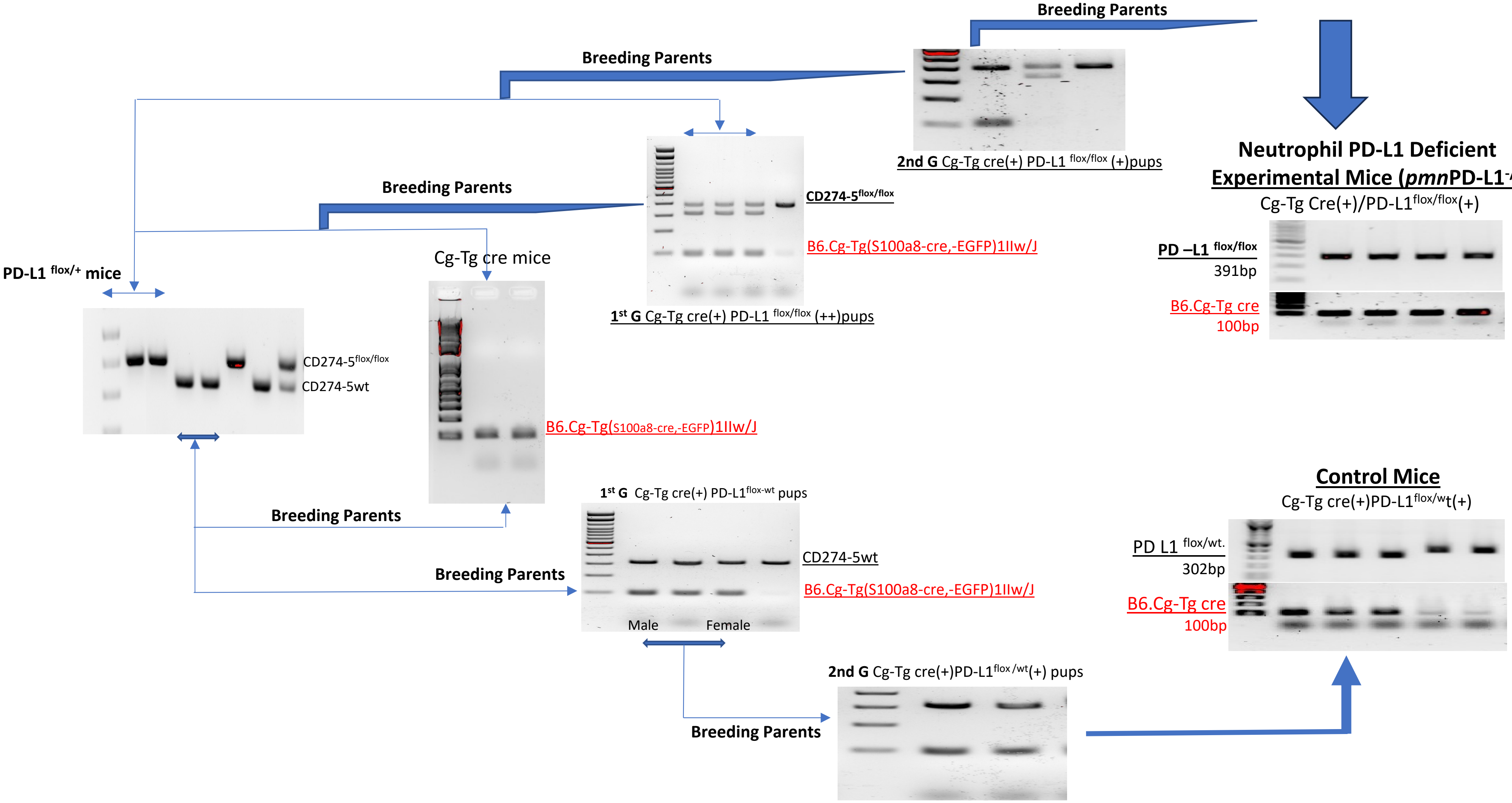

Supplement: Supplementary file 2 [file DataSheet2.pdf]
