# Supplementary material for: Endothelial cell, but not neutrophil, programmed cell death receptor-ligand 1 loss has a morbid impact on experimental murine shock/sepsis-induced lung injury
Source: Front Immunol. 2026 Jun 2;17:1816915. doi: 10.3389/fimmu.2026.1816915 (PMC13268903; doi:10.3389/fimmu.2026.1816915)

Supplemental Figure 3. The sequential model of Hem followed by CLP to induce indirect-ALI/ARDS like insult and temporal overview of experimental readouts assessed post Hem/CLP.

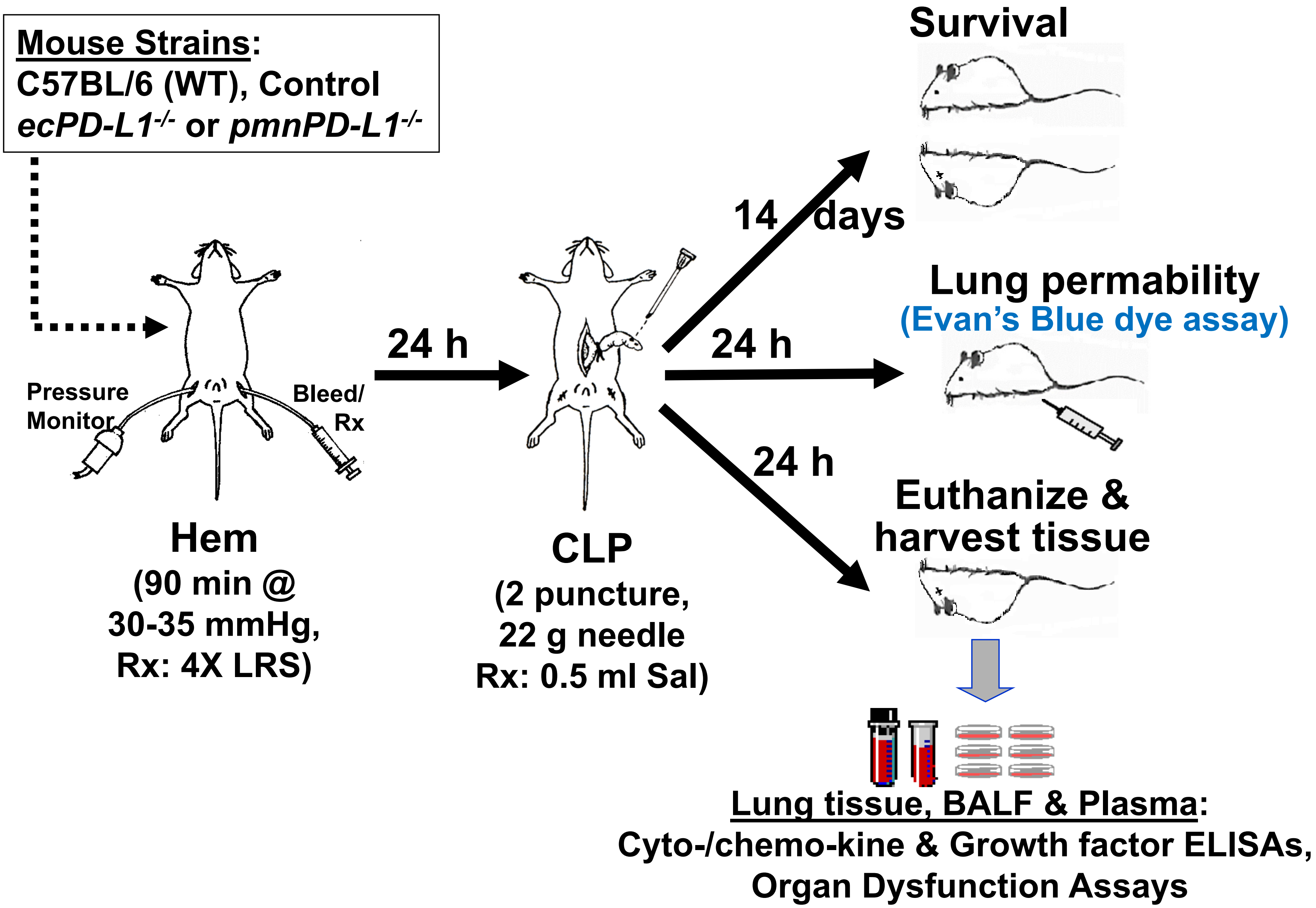

Supplement: Supplementary file 3 [file DataSheet3.pdf]
