# Supplementary material for: Endothelial cell, but not neutrophil, programmed cell death receptor-ligand 1 loss has a morbid impact on experimental murine shock/sepsis-induced lung injury
Source: Front Immunol. 2026 Jun 2;17:1816915. doi: 10.3389/fimmu.2026.1816915 (PMC13268903; doi:10.3389/fimmu.2026.1816915)

Supplemental Figure 4. C57BL/6 strain vs. Cre-Lox mouse construction 'Controls' from *ec*PD-L1$^{-/-}$ & *pmn*PD-L1$^{-/-}$ breeding, 14-day overall survival following exposure to the sequential insults of experimental shock sepsis were not statistically distinct. Control (from *ec*PD-L1$^{-/-}$ & *pmn*PD-L1$^{-/-}$ breeding process, respectively) or C57BL/6 mouse 14-day overall survival after subjecting them to the sequential insults of Hem/CLP as depicted by Kaplan-Meier curves. The n/treatment group is listed in parentheses; these groups were not significantly different as determined by a Log-Rank test.

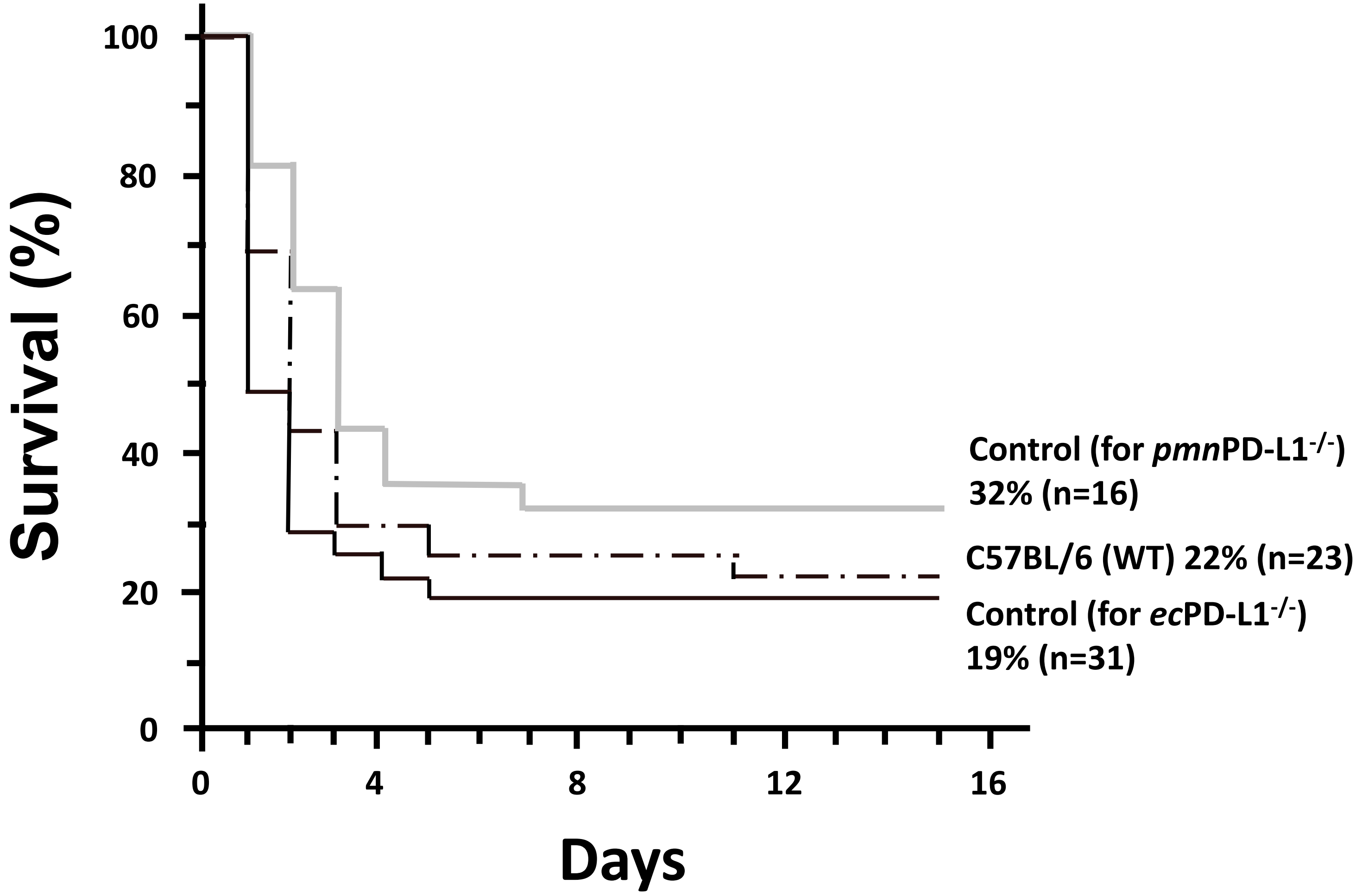

Supplement: Supplementary file 4 [file DataSheet4.pdf]
