# Supplementary material for: Endothelial cell, but not neutrophil, programmed cell death receptor-ligand 1 loss has a morbid impact on experimental murine shock/sepsis-induced lung injury
Source: Front Immunol. 2026 Jun 2;17:1816915. doi: 10.3389/fimmu.2026.1816915 (PMC13268903; doi:10.3389/fimmu.2026.1816915)
Supplement: Supplementary file 5 [file DataSheet5.pdf]

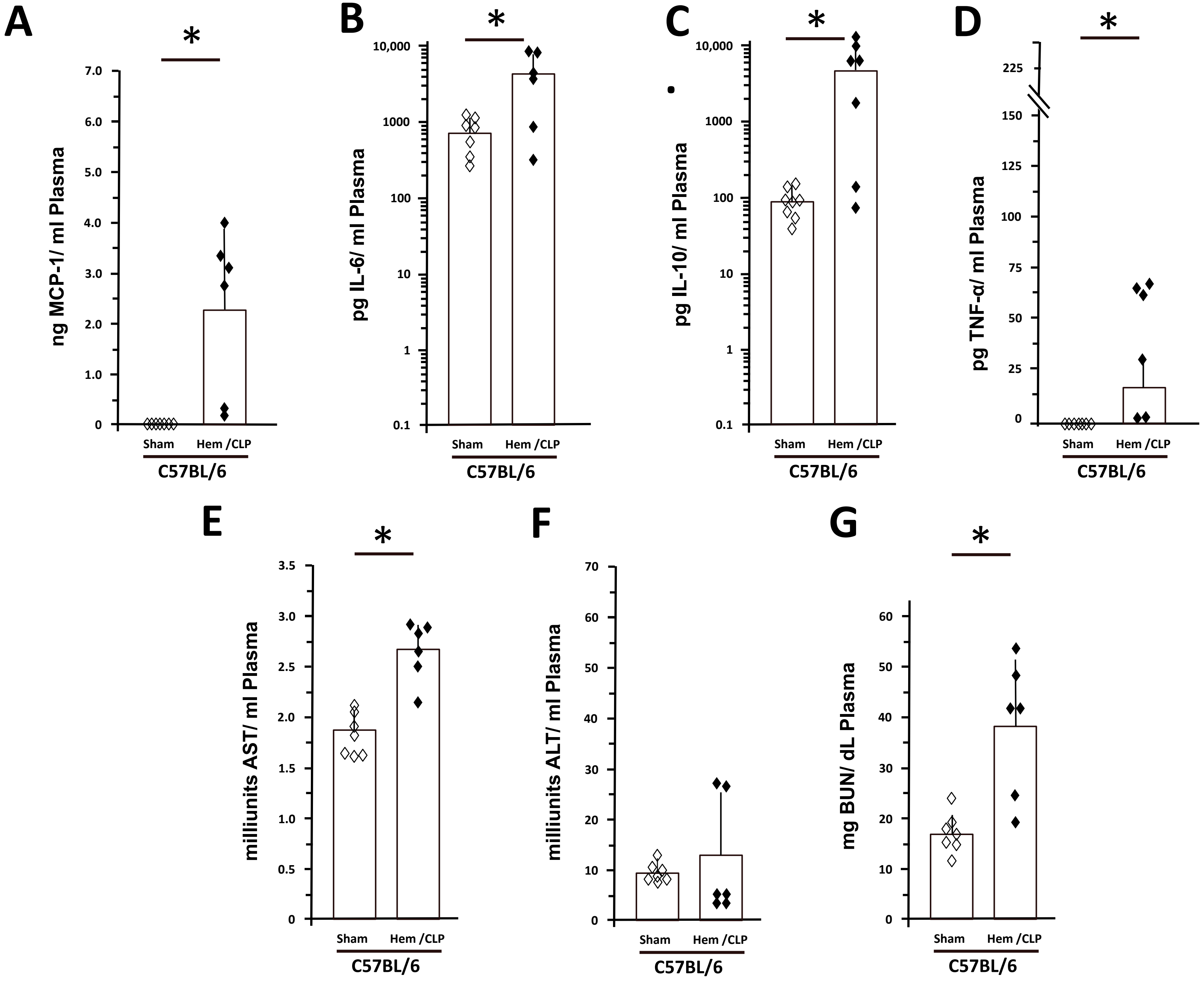


**Supplemental Figure 5. Hem/CLP typical induced marked changes in plasma chemokines/ cytokines and indices of organ injury, i.e., AST, ALT and BUN.** The assessment of systemic plasma chemokine/cytokine levels by ELISA or the AST, ALT and BUN concentration was determined by commercial assay. The n/treatment group are shown as symbols super-imposed on histogram depicting the group mean ± the standard deviation; the presence of a significant difference between groups was established at * $p<0.05$ with a Mann-Whittney-U test.
