# Supplementary material for: Endothelial cell, but not neutrophil, programmed cell death receptor-ligand 1 loss has a morbid impact on experimental murine shock/sepsis-induced lung injury
Source: Front Immunol. 2026 Jun 2;17:1816915. doi: 10.3389/fimmu.2026.1816915 (PMC13268903; doi:10.3389/fimmu.2026.1816915)
Supplement: Supplementary file 6 [file DataSheet6.pdf]

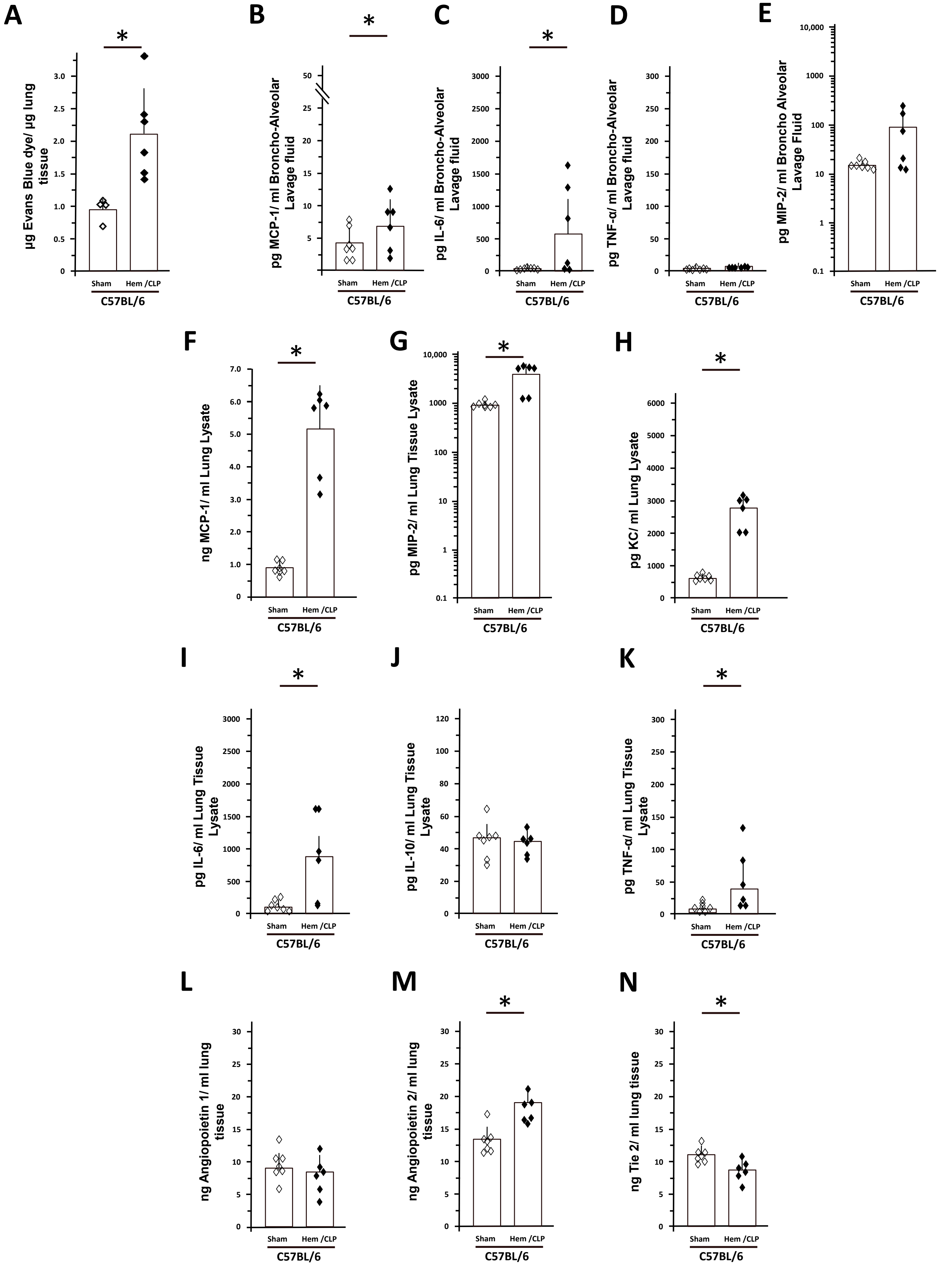

Supplemental Figure 6. Hem/CLP typical induced a marked changes in pulmonary vascular leak, BALF chemokines/cytokines, lung tissue chemokines/cytokines and lung tissues levels of Angiopoietin 1, 2 and Tie-2. The assessment of lung capillary leakage was done by extravasation of Evans blue (EB) dye method and the chemokine/cytokine or the Angiopoietin 1, 2 and Tie-2 levels were established by commercial ELISA. The n/treatment group are shown as symbols super-imposed on histogram depicting the group mean ± the standard deviation; The presence of a significant difference between groups was established at * $p<0.05$ with a Mann-Whittney-U test.
