# Supplementary material for: Endothelial cell, but not neutrophil, programmed cell death receptor-ligand 1 loss has a morbid impact on experimental murine shock/sepsis-induced lung injury
Source: Front Immunol. 2026 Jun 2;17:1816915. doi: 10.3389/fimmu.2026.1816915 (PMC13268903; doi:10.3389/fimmu.2026.1816915)
Supplement: Supplementary file 8 [file Table1.pdf]

Supplemental Table 1: PCR primer pairs

Mouse **PD-L1** gene:	sense, 5’-GAA GCT TTG CCT AAA GCA GG-3’
	antisense, 5’-GTC TGG AAA GAG CAG ACG AG-3’
	As described/designed by Taconic Biosciences Inc. (PD-L1^{flox} product: 391 bp; PD-L1^{WT} product: 320 bp)
Mouse **VE-Cadherin-Cre** transgene:	sense, 5′-GTG AAA CAG CAT TGC TGT CAC TT-3′
	antisense, 5′-GCG GTC TGG CAG TAA AAA CTA TC-3′
	As described/designed by The Jackson Laboratory. (Cre Transgene product: 100 bp ; Control product: 324 bp)
Mouse **S100a8-Cre** transgene:	sense, 5′-GTG AAA CAG CAT TGC TGT CAC TT-3′
	antisense, 5′-GCG GTC TGG CAG TAA AAA CTA TC-3′
	As described/designed by The Jackson Laboratory. (Cre Transgene product: 100 bp ; Control product: 324 bp)
